# Performance of the Xpert MTB/RIF assay for the diagnosis of pulmonary tuberculosis and rifampin resistance in a low-incidence, high-resource setting

**DOI:** 10.1371/journal.pone.0186139

**Published:** 2017-10-09

**Authors:** Jason P. Rice, Marva Seifert, Kathleen S. Moser, Timothy C. Rodwell

**Affiliations:** 1 Division of Preventive Medicine, University of California, San Diego, California, United States of America; 2 Division of Pulmonary, Critical Care and Sleep Medicine, University of California, San Diego, California, United States of America; 3 Tuberculosis Control and Refugee Health Program, County of San Diego Health and Human Services Agency, San Diego, California, United States of America; Wadsworth Center, UNITED STATES

## Abstract

Performance of the Xpert MTB/RIF assay, designed to simultaneously detect *Mycobacterium tuberculosis* complex (MTBC) and rifampin (RIF) resistance, has been well documented in low-resource settings with high TB-incidence. However, few studies have assessed its accuracy in low TB incidence settings. We evaluated the performance of Xpert MTB/RIF using clinical sputum specimens routinely collected from suspect pulmonary TB patients over a 4-year time period in San Diego County, California. Xpert MTB/RIF results were compared to acid-fast bacilli (AFB) smear microscopy, mycobacterial culture, and phenotypic drug susceptibility testing (DST). Of 751 sputum specimens, 134 (17.8%) were MTBC culture-positive and 2 (1.5%) were multidrug-resistant (MDR). For the detection of MTBC, Xpert MTB/RIF sensitivity was 89.6% (97.7% and 74.5% in smear-positive and -negative sputa, respectively) and specificity was 97.2%; while AFB smear sensitivity and specificity were 64.9% and 77.8%, respectively. Xpert MTB/RIF detected 35 of 47 smear-negative culture-positive specimens, and excluded 124 of 137 smear-positive culture-negative specimens. Xpert MTB/RIF also correctly excluded 99.2% (121/122) of nontuberculous mycobacteria (NTM) specimens, including all 33 NTM false-positives by smear microscopy. For the detection of RIF resistance, Xpert MTB/RIF sensitivity and specificity were 100% and 98.3%, respectively. Our findings demonstrate that Xpert MTB/RIF is able to accurately detect MTBC and RIF resistance in routinely collected respiratory specimens in a low TB-incidence setting, with comparable performance to that achieved in high-incidence settings; and suggest that under these conditions the assay has particular utility in detecting smear-negative TB cases, excluding smear-positive patients without MTBC disease, and differentiating MTBC from NTM.

## Introduction

Despite declining global incidence and mortality, tuberculosis (TB) remains a leading cause of death among infectious diseases worldwide, with an estimated 10.4 million new cases and 1.4 million deaths in 2015 [1,2]. In the clinical management of pulmonary TB, rapid and accurate detection of *Mycobacterium tuberculosis* complex (MTBC) and drug-resistance is essential. Likewise on a population scale, early case detection and appropriate treatment are the most effective control strategies to reduce TB transmission [2,3].

Acid-fast bacilli (AFB) smear microscopy is a relatively simple and inexpensive screening tool, however it is limited by poor sensitivity, detecting only 50–70% of culture-positive TB cases [4,5]. Smear microscopy is unable to distinguish MTBC from nontuberculous mycobacteria (NTM), or viable from nonviable organisms, and thus has the potential for false-positives and low positive predictive value (PPV). In addition, smear microscopy is unable to differentiate drug resistant and susceptible strains of MTBC [6,7].

Mycobacterial culture is considered the reference standard for the detection of MTBC. It is critical in the diagnosis of smear-negative TB, estimated to comprise 35% of the world’s TB cases, as well as monitoring patient response to treatment and the adequacy of TB control activities [3,7,8]. However, culture is time consuming and resource-intensive, requiring up to 6 and 8 weeks for final identification on liquid and solid culture media, respectively [6,9].

Culture-based phenotypic drug susceptibility testing (DST) is generally regarded as the reference standard for drug resistant (DR) TB detection, however on average it requires an additional 1–3 and 4–6 weeks for results on liquid and solid culture, respectively [7,9,10]. Also, recent evidence suggests that phenotypic DST may fail to detect some low-level but clinically significant DR TB strains [11–15].

Compared with conventional TB diagnostics, molecular techniques are able to directly detect MTBC and drug resistance with high accuracy and improved efficiency, and thus have become increasingly emphasized in TB control policy [7,16,17]. Commercially available nucleic acid amplification tests (NAATs) are able to detect MTBC and rifampin (RIF) resistance (alone or in combination with isoniazid [INH] resistance) weeks earlier than culture and phenotypic DST, with comparable accuracy [14,18–20].

The Xpert MTB/RIF assay (Cepheid, Sunnyvale, CA) is a novel, automated cartridge-based NAAT capable of simultaneously detecting MTBC and RIF resistance within 2 hours; endorsed by the WHO in 2010, approved by the FDA in 2013, and widely regarded as a breakthrough in TB diagnostics [21–25]. The assay is performed on the Cepheid GeneXpert multi-disease instrument system, which integrates sample purification, nucleic acid amplification, and detection of target sequences. Xpert MTB/RIF uses hemi-nested real-time PCR for the detection of MTBC and RIF-resistant (RIF^r^) strains, using three primers to amplify the MTBC-specific sequence of the *rpoB* gene, and five molecular probes to detect mutations within the gene’s RIF resistance-determining region (RRDR) [21,26,27]. The assay can be performed directly on raw sputum or concentrated sediments. Specimens are liquefied and deactivated by a mycobactericidal sample reagent, and after cartridge loading all steps are fully automated and self-contained [21,27].

Several large multi-center studies and meta-analyses have evaluated the clinical performance of Xpert MTB/RIF in the diagnosis of pulmonary TB and RIF resistance [28–32]. In respiratory specimens the assay has demonstrated excellent accuracy for the detection of MTBC and RIF resistance, substantially increased case detection over AFB smear microscopy, and the ability to differentiate MTBC from NTM. However, the majority of Xpert MTB/RIF performance evaluations have been carried out in resource-poor regions of the world with high TB-incidence, where case rates may exceed 900 per 100,000 persons [32]. In the United States and other low-incidence countries (<10 cases per 100,000) [33], data is lacking [34–41]. In addition to being limited in number and size, studies in these settings used differing Xpert MTB/RIF testing protocols, and were conducted under a variety of research conditions.

Our aim was to evaluate the accuracy of the Xpert MTB/RIF assay in the diagnosis of pulmonary TB and RIF resistance in a low TB-incidence setting, as applied in the course of routine clinical practice. The results of this study have the potential to clarify the role of this application in TB clinical management, and to inform future testing protocols in low-incidence, high-resource settings.

## Materials and methods

This study was conducted at the San Diego County Public Health Department, using data and specimens routinely collected by the microbiology section of the San Diego County Public Health Laboratory (SDCPHL). SDCPHL receives and processes clinical specimens from the county TB Clinic as well as other local medical treatment facilities, and routinely performs AFB smear microscopy, mycobacterial culture, phenotypic DST, and NAA testing. In cases of suspected DR TB, SDCPHL also routinely sends specimens to the California Department of Public Health (CDPH) Microbial Diseases Laboratory (MDL) for pyrosequencing (PSQ).

San Diego County has an annual TB case rate of 7.3 per 100,000, with 234 TB cases reported in 2015 [42]. Annually, approximately 18% of reported TB cases are resistant to at least one first-line anti-TB drug, and 1.1% have MDR TB [43,44].

### Study design and specimens

In this retrospective study, all sputum specimens collected from patients in San Diego County that were tested using the Xpert MTB/RIF assay at the SDCPHL from Jan 2012 to Jan 2016 were included in the study. Specimens were collected from patients at the San Diego County TB Clinic and other county medical treatment facilities, in the course of routine clinical evaluations for pulmonary TB. At the SDCPHL, in addition to Xpert MTB/RIF testing each specimen underwent routine AFB smear microscopy and mycobacterial culture; and isolates from each MTBC culture-positive specimen underwent phenotypic DST. All corresponding test results were available for each specimen, and no specimens were excluded from the study.

Other variables obtained for each specimen included: patient age, dates of collection and reporting, and method of specimen collection (induced *vs* expectorated sputa). In addition, TB Control surveillance data on treatment status were obtained for all Xpert-positive culture-negative samples, and results of PSQ performed by the CDPH MDL were obtained for all culture-positive samples with RIF resistance detected by phenotypic DST or Xpert MTB/RIF.

### AFB smear microscopy

Specimens were processed using the N-acetyl-L-cysteine-NaOH (NALC-NaOH) method for digestion and decontamination. Specimens were concentrated by centrifugation at 3,200 Xg for 20 min, and sediments were reconstituted with approximately 2.0 ml of 0.067 M sterile phosphate buffer (pH 6.8). Smear microscopy was performed on processed sediments using auramine-O fluorochrome staining, and confirmed with Ziehl-Neelsen (ZN) staining. Smear-positive specimens were graded from 1+ to 4+ according to CDC guidelines [6].

### Mycobacterial culture

Processed sputum sediments from each sample were inoculated into Middlebrook 7H10 solid slants, as well as liquid broth Mycobacterial Growth Indicator Tubes (MGIT; Becton Dickinson [BD], Sparks, MD) using the BD BACTEC MGIT 960 automated system. Liquid and solid cultures were incubated at 37°C for up to 6 and 8 weeks, respectively. Positive mycobacterial isolates were confirmed with ZN staining, and species identification was performed with conventional biochemical tests, observation of colony morphology, and/or nucleic acid hybridization using molecular probes (Accuprobe; Gen-Probe, San Diego, CA). Niacin accumulation and nitrate reduction biochemical identification tests were performed on Lowenstein-Jensen (LJ) solid culture media, inoculated from 7H10 or MGIT isolates with positive mycobacterial growth.

### Drug susceptibility testing

All MTBC isolates underwent indirect phenotypic first-line DST using the liquid BACTEC MGIT 960 system, according to the manufacturer’s recommendations [45,46]. First-line anti-TB agents tested for resistance included RIF, INH, ethambutol (EMB) and pyrazinamide (PZA). Susceptibilities were tested at the following standard critical concentrations: RIF, 1.0 μg/ml; INH, 0.1 μg/ml and 0.4 μg/ml; EMB, 5.0 μg/ml; PZA, 100 μg/ml.[6] PSQ was performed by the CDPH MDL using the PyroMark Q96ID system (Qiagen, Valencia, CA) to sequence molecular targets associated with resistance to RIF, INH, fluoroquinolones and anti-TB injectable agents, as has been previously described [14,47,48].

### Xpert MTB/RIF assay

The Xpert MTB/RIF assay was run on the GeneXpert Dx instrument system according to the manufacturer’s recommendations [27]. Briefly, after digestion, decontamination and concentration, 0.5 ml of re-suspended sediment was transferred to a conical screw-capped tube, 1.5 ml of Xpert MTB/RIF sample reagent was added by sterile pipette, and the tube was recapped and shaken vigorously 10–20 times. The sample was incubated for a total of 15 minutes at 20–30°C, with manual agitation 10–20 times at one point between 5 and 10 minutes into the incubation period. The reagent-treated sample was then transferred by sterile pipette into the sample chamber of the Xpert MTB/RIF cartridge and loaded into the GeneXpert Dx instrument system for sample processing. In the event of “no result”, “invalid” or “error” results, the test was repeated according to the manufacturer’s recommendations using a new Xpert MTB/RIF cartridge.

The SDCPHL and TB Control Program employed the Xpert MTB/RIF assay in accordance with CDC recommendations for NAA testing: to perform on at least one respiratory specimen, preferably the first collected, from each patient with signs and symptoms of pulmonary TB and for whom the test result would alter case management or TB control activities [16,49]. Some variation in protocol existed during the 4-year study time period due to resource availability, however in general the Xpert MTB/RIF assay was used to reflexively test the first AFB smear-positive specimen collected from each patient, as well as AFB smear-negative specimens per clinician request in patients with a high suspicion of pulmonary TB.

### Statistical analysis

Sensitivity, specificity, positive predictive value (PPV), negative predictive value (NPV), and positive and negative likelihood ratios (LRs) were calculated for the detection of MTBC and RIF resistance using culture and phenotypic DST as the reference standards, respectively, with exact Clopper-Pearson 95% confidence intervals. Final test results were available for all specimens, and all were included in the analyses. Standard descriptive statistics were used to characterize the study population. Statistical analyses were performed using IBM SPSS Statistics, version 23.0 (IBM Corp., Armonk, NY) and GraphPad Prism, version 6.0 (GraphPad Software, La Jolla, CA).

This study was reviewed and certified as exempt by the institutional review board of the University of California San Diego, and all samples were accessed anonymously. In addition the study was reviewed and received approval from the County of San Diego Health and Human Services Strategic Planning and Operational Support Division.

## Results

A total of 751 sputum specimens were included in the study, of which 653 (87%) were induced, and the remainder expectorated (Table 1). Specimens were collected from 637 patients (median age, 50 [IQR 35–60]), of which 545 (85.6%) contributed 1 specimen, 74 (11.6%) contributed 2 specimens, and 16 (2.5%) contributed 3 specimens. One hundred thirty-four (17.8%) specimens were MTBC culture-positive, including *M*. *tuberculosis* (n = 132) and *M*. *bovis* (n = 2); while 495 (65.9%) specimens were culture-negative for mycobacteria, and 122 (16.3%) yielded NTM on culture. Phenotypic first-line DST results were available for all 134 MTBC culture-positive specimens, with 2 (1.5%) of specimens identified as MDR, and the following numbers of resistant specimens in total: RIF^r^ (n = 2), INH^r^ (n = 14, at 0.1 μg/ml; n = 10, at 0.4 μg/ml), PZA^r^ (n = 4), EMB^r^ (n = 2).

**Table 1:** Specimen (n = 751) and patient (n = 637) characteristics.

Characteristic	No.	%
Sputum collection method		
Induced	653	87.0
Expectorated	98	13.0
Mycobacterial culture results		
MTBC	134	17.8
NTM	122	16.3
Negative	495	65.9
AFB smear results		
Positive	224	29.8
Negative	527	70.2
DST results (n = 134)		
RIF^r^ (1.0 μg/ml)	2	1.5
INH^r^ (0.1 μg/ml)	14	10.4
INH^r^ (0.4 μg/ml)	10	7.5
PZA^r^ ((100 μg/ml)	4	3.0
EMB^r^ (5.0 μg/ml)	2	1.5
Specimens per patient		
One	545	85.6
Two	74	11.6
Three	16	2.5
Five	2	0.3
Median patient age in years (IQR)	50	(35–60)

Abbreviations: MTBC, *Mycobacterium tuberculosis* complex; NTM, Nontuberculous mycobacteria; AFB, acid-fast bacilli; DST, drug susceptibility testing; RIF, rifampin; INH, isoniazid; PZA, pyrazinamide; EMB, ethambutol; ^*r*^, resistant; IQR, interquartile range

### Detection of Mycobacterium tuberculosis complex

Performance of the Xpert MTB/RIF assay for the detection of MTBC overall and stratified by smear status is shown in Table 2, using culture as the reference standard and in comparison with AFB smear microscopy. Xpert MTB/RIF detected 89.6% (120/134) of MTBC culture-positive specimens overall, including both *M*. *bovis* specimens. Sensitivity was 97.7% (85/87) among smear-positive sputa, and 74.5% (35/47) among smear-negative. Of the 14 Xpert-negative culture-positive specimens, 12 were smear-negative and 2 were 1+ smear-positive; sensitivity was 91.3% (21/23) among 1+ specimens and 100% (64/64) among all other smear-positives. Xpert MTB/RIF specificity was 97.2% (600/617) overall; 90.5% (124/137) among smear-positive sputa, and 99.2% (476/480) among smear-negative. Additionally, sensitivity and specificity analyses were performed on a subset of results which excluded multiple specimens per patient, yielding nearly identical results to analyses with inclusion of all study samples (data available for review).

**Table 2:** Performance of Xpert MTB/RIF for the detection of MTBC as stratified by smear status, using mycobacterial culture as the reference standard; In comparison with the performance of AFB smear microscopy.

			Culture results, No.		Total	Performance, % (95% CI)^a^						
			MTBC+	MTBC-		Sensitivity	Specificity	PPV	NPV	LR+	LR-	MTBC%^b^
Xpert results, No.												
Overall		+	120	17	137	89.6	97.2	87.6	97.7	32.5	0.11	17.8
		-	14	600	614	(83.1–94.2)	(95.6–98.4)	(80.9–92.6)	(96.2–98.8)			
		Total	134	617	751							
Smear positive		+	85	13	98	97.7	90.5	86.7	98.4	10.3	0.03	38.8
		-	2	124	126	(91.9–99.7)	(84.3–94.9)	(78.4–92.7)	(94.4–99.8)			
		Total	87	137	224							
Smear negative		+	35	4	39	74.5	99.2	89.7	97.5	89.4	0.26	8.9
		-	12	476	488	(59.7–86.1)	(97.9–99.8)	(75.8–97.1)	(95.7–98.7)			
		Total	47	480	527							
Amended^c^	Overall	+	120	2	122	89.6	99.7	98.4	97.7	269.6	0.10	18.2
		-	14	600	614	(83.1–94.2)	(98.8–100)	(94.2–99.8)	(96.2–98.8)			
		Total	134	602	736							
	Smear positive	+	85	0	85	97.7	100	100	98.4	N/A	0.02	41.2
		-	2	124	126	(91.9–99.7)	(97.1–100)	(95.6–100)	(94.4–99.8)			
		Total	87	124	211							
	Smear negative	+	35	2	37	74.5	99.6	94.6	97.5	178.0	0.26	9.0
		-	12	476	488	(59.7–86.1)	(98.5–100)	(81.8–99.3)	(95.7–98.7)			
		Total	47	478	525							
AFB Smear results, No.												
		+	87	137	224	64.9	77.8	38.8	91.1	2.92	0.45	17.8
		-	47	480	527	(56.2–73.0)	(74.3–81.0)	(32.4–45.6)	(88.3–93.4)			
		Total	134	617	751							

Abbreviations: MTBC, *Mycobacterium tuberculosis* complex; AFB; acid-fast bacilli; CI, confidence interval; PPV, positive predictive value; NPV, negative predictive value; LR+, positive likelihood ratio; LR-, negative likelihood ratio.

^*a*^LR+ and LR- presented as ratio measures.

^*b*^Prevalence of MTBC culture-positive specimens.

^*c*^Excluding culture-negative specimens from patients receiving anti-TB treatment at the time of testing (n = 15).

Of the 17 Xpert-positive culture-negative specimens, TB Control Program surveillance data revealed that 15 specimens were collected from patients receiving anti-TB treatment at the time of testing, including all 13 smear-positives. As detailed in Table 2, excluding these 15 specimens from the analysis, Xpert MTB/RIF specificity increased to 99.7% (600/602) overall; 100% (124/124) among smear-positive sputa and 99.6% (476/478) among smear-negative. Additionally, PPV increased to 98.4% (120/122) overall; 100% (85/85) among smear-positive sputa, and 94.6% (35/37) among smear-negative.

AFB smear microscopy demonstrated a sensitivity of 64.9% (87/134) and specificity of 77.8% (480/617) for the detection of MTBC, referent to culture (Table 2). PPV and NPV for AFB smear microscopy were 38.8% (87/224) and 91.1% (480/527), respectively. Of the 47 smear-negative culture-positive specimens that microscopy failed to detect, 35 were correctly detected by Xpert MTB/RIF. Of the 137 smear-positive culture-negative specimens that microscopy identified as MTBC, 124 were correctly excluded by Xpert MTB/RIF.

Stratified by sputum collection method, Xpert MTB/RIF sensitivity and specificity were 89.3% (100/115) and 97.2% (526/538) among induced sputum samples, and 90.9% (20/22) and 97.4% (74/76) among expectorated sputum samples, respectively.

The prevalence of culture-confirmed NTM among all specimens was 16.3% (122/751), of which Xpert MTB/RIF correctly excluded all but 1 of 122 specimens (Table 3). Xpert MTB/RIF specificity and NPV for the exclusion of NTM were 99.2% (121/122) and 89.6% (121/135), respectively. In contrast, AFB smear microscopy falsely identified 33 NTM-containing specimens as MTBC, with a specificity of 73% (89/122) and NPV of 65.4% (89/136). Of the 137 total specimens falsely identified as MTBC by AFB smear microscopy, 24.1% (33/137) were NTM culture-confirmed.

**Table 3:** Performance of Xpert MTB/RIF vs AFB smear microscopy for the exclusion of NTM, using mycobacterial culture as the reference standard.

		Culture results, No.			Total	Performance, % (95% CI)^a^		
			MTBC-					
		MTBC+	NTM	Neg^b^		Specificity	NPV	NTM%^c^
Xpert results, No.	+	120	1	16	137	99.2	89.6	16.3
	-	14	121	479	614	(95.5–100)	(83.2–94.2)	
	Total	134	122	495	751			
AFB Smear results No.	+	87	33	104	224	73.0	65.4	
	-	47	89	391	527	(64.2–80.6)	(56.8–73.4)	
	Total	134	122	495	751			

Abbreviations: NTM, Nontuberculous mycobacteria; MTBC, *Mycobacterium tuberculosis* complex; AFB; acid-fast bacilli; CI, confidence interval; NPV, negative predictive value.

^*a*^Specificity and NPV with regard to NTM.

^*b*^Negative for mycobacteria.

^*c*^Prevalence of NTM among all specimens.

### Detection of rifampin resistance

RIF resistance was identified by phenotypic DST in 2 (1.5%) of the 134 MTBC culture-positive specimens; both of which were MDR, and resistant to all 4 first-line anti-TB agents tested (RIF, INH, PZA and EMB). The Xpert MTB/RIF assay reported RIF resistance in both specimens identified as RIF^r^ by phenotypic DST, and also reported RIF resistance in 2 MTBC specimens that DST identified as RIF-susceptible (RIF^s^). Of the 14 MTBC culture-positive specimens that Xpert MTB/RIF failed to detect, all were identified as RIF^*s*^ by DST. Using phenotypic DST as the reference standard, Xpert MTB/RIF sensitivity and specificity for the detection of RIF resistance were 100% (2/2) and 98.3% (116/118), respectively (Table 4).

**Table 4:** Performance of Xpert MTB/RIF for the detection of RIF resistance, relative to phenotypic DST.

		DST results, No.			Performance, % (95% CI)^b^						
		RIF-R	RIF-S	Total	Sensitivity	Specificity	PPV	NPV	LR+	LR-	RIF-R%^c^
Xpert results, No.											
	RIF-R	2	2	4	100	98.3	50	100	59	0	1.5
	RIF-S	0	116	116	(15.8–100)	(94.0–99.8)	(6.8–93.2)	(96.9–100)			
	MTB-	0	14	14							
	Total	2	132	134							
Discrepant resolution^a^	RIF-R	3	1	4	100	99.2	75	100	117	0	2.2
	RIF-S	0	116	116	(29.2–100)	(95.3–100)	(19.4–99.4)	(96.9–100)			
	MTB-	0	14	14							
	Total	3	131	134							

Abbreviations: RIF, rifampin; DST, drug susceptibility testing; -R, resistant; -S, susceptible; MTB-, negative for *Mycobacterium tuberculosis* complex; CI, confidence interval; PPV, positive predictive value; NPV, negative predictive value; LR+, positive likelihood ratio; LR-, negative likelihood ratio.

^*a*^Of 2 specimens with discrepant results (RIF-R by Xpert and RIF-S by phenotypic DST), 1 specimen is reclassified as RIF-R based on DNA sequencing detection of *rpoB* mutation (CTG→CCG Leu511Pro) associated with low-level but clinically significant RIF resistance.

^*b*^LR+ and LR- presented as ratio measures.

^*c*^Prevalence of RIF-R specimens among all MTBC culture-positive specimens.

Of the 2 specimens with discrepant Xpert MTB/RIF and phenotypic DST results, DNA sequencing revealed 1 specimen to have an *rpoB* gene mutation (CTG→CCG Leu511Pro) reported by the CDPH MDL to be associated with low-level but clinically significant RIF resistance. In the other discrepant isolate sequencing detected a silent *rpoB* mutation (TTC→TTT 514Phe) that is not known to confer RIF resistance. For the 2 specimens with concordant Xpert MTB/RIF and phenotypic DST RIF^*r*^ results, sequencing confirmed RIF resistance-conferring mutations (TCG→TTG Ser531Leu) in both isolates. Incorporating sequencing results for discrepancy resolution, Xpert MTB/RIF specificity and PPV increased to 99.2% (116/117) and 75% (3/4), respectively (Table 4). The Xpert MTB/RIF assay also reported RIF resistance in 1 culture-negative specimen. This was one of the 15 Xpert-positive culture-negative specimens collected from patients on anti-TB treatment, and review of surveillance data revealed a silent *rpoB* mutation reported on a previously sequenced isolate from this patient.

Among all MTBC culture-positive specimens, phenotypic DST identified a total of 16 specimens with resistance to any first-line anti-TB agent, as detailed in Table 5. Correcting for the discrepant RIF^s^ phenotypic DST result, 8.2% (11/134) of MTBC specimens were INH-monoresistant, 1.5% (2/134) of MTBC specimens (and 100% [2/2] of *M*. *bovis* specimens) were PZA-monoresistant, and 2.2% (3/134) of MTBC specimens were MDR; of which 2 were resistant to RIF, INH, PZA and EMB, and 1 was resistant to RIF and INH only.

**Table 5:** Drug resistant specimens by phenotypic DST (n = 16).

Specimen	Anti-TB agent				
	RIF	INH^a^	INH^b^	PZA	EMB
1	S	R	R	S	S
2	R	R	R	R	R
3	S^c^	R	R	S	S
4	S	R	R	S	S
5	S	S	S	R^e^	S
6	S	R	R	S	S
7	S	R	S	S	S
8	S	R	S	S	S
9	S	R	R	S	S
10	S	R	R	S	S
11	S	R	R	S	S
12	R	R	R	R	R
13	S	S	S	R^e^	S
14	S	R	R	S	S
15	S^d^	R	S	S	S
16	S	R	S	S	S

Abbreviations: RIF, rifampin; INH, isoniazid; PZA, pyrazinamide; EMB, ethambutol; R, resistant; S, susceptible.

^*a*^Critical concentration (0.1 μg/ml).

^*b*^Critical concentration (0.4 μg/ml).

^*c*^RIF resistance-conferring mutation on sequencing (CTG→CCG Leu511Pro).

^*d*^Silent mutation on sequencing (TTC→TTT 514Phe).

^*e*^*Mycobacterium bovis* isolate.

## Discussion

Accuracy of the Xpert MTB/RIF assay for the diagnosis of pulmonary TB and RIF resistance assay has been previously described, primarily in studies conducted in resource-poor countries of the world with high TB incidence [28–32]. In respiratory specimens the assay has demonstrated a pooled sensitivity of 89% (98% and 67% among smear-positive and–negative specimens, respectively) and specificity of 99% for the detection of MTBC, and pooled sensitivity and specificity of 95% and 98% for the detection of RIF resistance, respectively [32]. In this study we evaluated the performance of Xpert MTB/RIF in routine clinical practice over a 4-year time period in San Diego County, a low TB-incidence setting with an annual case rate of 7.3 per 100,000 [42]. Our results compare favorably with pooled data from global studies, and demonstrate the assay’s ability to perform equally well in a low TB-incidence setting.

Xpert MTB/RIF and other NAATs have consistently shown high sensitivity among smear- and culture-positive specimens, however their variable sensitivity in smear-negative specimens has raised concern [16,19]. This test characteristic is of particular importance in low TB-incidence settings, where it is estimated that up to 60% of TB cases are smear-negative [33]. An Xpert MTB/RIF study performed in Canada (4.6 cases per 100,000) reported a sensitivity of 28% in smear-negative sputum specimens, and suggested the assay may have limited utility in low-incidence, high-resource countries [41]. In the present study, despite an MTBC prevalence of only 8.9% (47/527) among smear-negative specimens, Xpert MTB/RIF correctly detected 74.5% (35/47) of smear-negative culture-positive specimens, with a specificity of 99.2%. This performance was superior to the pooled meta-analyses, and suggests that for the detection of smear-negative MTBC, the assay has utility in both high- and low TB-incidence settings.

Results from our study illustrate the marked improvement in case detection that Xpert MTB/RIF offers over AFB smear microscopy (sensitivity and specificity of 64.9% and 77.8%, respectively). Of 134 culture-positive specimens in this study, 47 (35.1%) were smear-negative. Among those MTBC specimens missed by smear microscopy, 35 were correctly detected by Xpert MTB/RIF, increasing overall case detection by 26.1% (35/134). Perhaps more importantly for U.S. TB programs to consider, however, is that microscopy falsely identified 137 MTBC culture-negative specimens, of which 124 (90.5%) were correctly excluded by Xpert MTB/RIF. In this setting the assay appears to have a significant advantage over AFB smear microscopy, not only in detecting smear-negative TB cases, but also ruling out smear-positive individuals without MTBC disease.

NAATs, Xpert MTB/RIF included, have proven especially useful in settings with high NTM-burden, as they are able to rapidly differentiate MTBC from NTM and potentially avoid unnecessary treatment [19,50,51]. This is relevant in the U.S. where NTM- exceeds TB-burden, and evidence suggests that NTM infections are increasing by approximately 8% annually, with clinically significant pulmonary NTM infections increasing in particular [52,53]. The prevalence of NTM in our study was 16.3% (122/751), of which Xpert MTB/RIF correctly excluded 99.2% (121/122). In contrast, AFB smear microscopy falsely identified 33 NTM specimens as MTBC. These false-positives represented 24.1% (33/137) of smear-positive culture-negative specimens, and Xpert MTB/RIF correctly excluded them all. With these performance characteristics, the assay appears to have particular utility in this low TB-incidence setting with high NTM-burden.

The Xpert MTB/RIF assay reported 14 false-negative and 17 false-positive results in this study, referent to culture. Of the 14 Xpert-negative culture-positive specimens, 12 were smear-negative and 2 were 1+ smear-positive. As has been described previously, this can likely be attributed to low bacillary load, given that the Xpert MTB/RIF reported limit of detection (LOD) in sputum (131 cfu/mL) is higher than culture (10–100 cfu/mL) [6,24]. Of the 17 Xpert-positive culture-negative specimens, 15 were collected from patients on anti-TB drugs at the time of testing. The Xpert MTB/RIF assay is not intended for use in monitoring response to treatment, as it has the potential to detect genetic material from remnant, non-viable organisms [26,27]. This was likely the cause of the false-positives observed in our study. In clinical practice, situations may prompt clinicians to use the assay in this manner (eg., upon collection of a smear-positive specimen while monitoring treatment response in an established, previously culture-converted TB case); and given the intent of this study was to evaluate the assay’s performance under routine practice conditions, these specimens were included. However, our findings demonstrate its limited utility in these circumstances, and reinforce that Xpert MTB/RIF results must be questioned when testing patients on anti-TB drugs. Additionally, secondary analysis with exclusion of these specimens revealed substantially improved specificity (99.7%) and PPV (98.4%), in particular among smear-positive sputa (specificity and PPV of 100%), illustrating the assay’s superior performance when used as recommended [15,27,49].

Consistent with the incidence of MDR TB in the U.S. and San Diego County, in our study phenotypic DST identified MDR TB in 1.5% (2/134) of MTBC isolates, which included all RIF^r^ strains identified [43,44]. Xpert MTB/RIF detected both RIF^*r*^ (MDR) specimens, and correctly identified 116 RIF^*s*^ specimens. Referent to phenotypic DST, for the detection of RIF resistance (and consequently MDR TB), Xpert MTB/RIF sensitivity and specificity were 100% and 98.3%, respectively. In this study, in a setting with a low incidence of RIF resistance and MDR TB, these results compare favorably with global pooled meta-analysis findings.

Prior research has raised concern over the specificity of Xpert MTB/RIF for the detection of RIF^r^ strains relative to phenotypic DST. However, given accumulating evidence that the reference standard may fail to detect some low-level but potentially clinically relevant RIF resistance, it appears that Xpert MTB/RIF specificity may be underestimated [11,15]. This evidence comes from a number of studies comparing solid and liquid culture-based phenotypic DST with *rpoB* gene sequencing, demonstrating that MTBC strains with specific *rpoB* mutations (e.g., CTG→CCG Leu511Pro, GAC→TAC Asp516Tyr, CAC→CTC His526Leu, CAC→AAC His526Asn, CTG→CCG Leu533Pro) almost always appear susceptible on MGIT DST. Of note, while the majority of these “disputed” mutations are located within the RRDR of the *rpoB* gene (codons 507 to 533), some of the most common (Leu511Pro and Leu533Pro) are located at the extreme ends of the RRDR [11–14]. Silent RRDR mutations that do not result in an amino acid change and do not confer resistance have also been documented (eg., TTC→TTT 514Phe), however these appear to be rare [11,14,54,55]. Reviewing an original study on Xpert MTB/RIF performance [28], Van Deun et al. note that sequencing revealed RRDR mutations in all 9 discordant phenotypically susceptible strains, of which only 1 silent mutation was truly a false-positive, since all others were low MIC mutations not detectable on MGIT DST [11].

In the present study, of the 2 specimens with discrepant results (RIF^*r*^ by Xpert MTB/RIF and RIF^*s*^ by phenotypic DST), sequencing revealed 1 isolate with a commonly missed and potentially clinically relevant mutation (CTG→CCG Leu511Pro), and the other with a silent mutation (TTC→TTT 514Phe). Using sequencing for discrepancy resolution, Xpert MTB/RIF specificity for the detection of RIF resistance (and consequently MDR TB) increased from 98.3% to 99.2%, and PPV increased from 50% to 75%. Evidence suggests that while the assay has high NPV in all settings (>99%), PPV is dependent on the prevalence of RIF resistance, ranging from <70% to >90% in settings where the underlying prevalence is <5% and >15%, respectively [15]. Our results are compatible with these estimates, given the low prevalence of RIF resistance in this study, and historically in San Diego County (<2%) [56]. Considering the potential for false-positives and low PPV with Xpert MTB/RIF, our findings reinforce CDC recommendations that all RIF^*r*^ results prompt rapid DNA sequencing for first- and second-line anti-TB drugs, in order to tailor MDR treatment to the patient’s specific resistance profile [49].

In the U.S., working under general CDC guidance for NAA testing, TB programs have been challenged to determine the most effective and efficient use of Xpert MTB/RIF in their practice settings. The assay has been incorporated into a variety of testing algorithms (e.g., testing smear-positive specimens only *vs* smear-positive and high-suspicion smear-negative specimens *vs* testing specimens from all suspected cases), yet no one approach has gained consensus. In a study of Xpert MTB/RIF cost-effectiveness in the U.S., Choi et al. proposed that the addition of the assay would result in lower total health care costs compared to algorithms using microscopy and culture alone, and that testing at least one sputum specimen from all patients with signs/symptoms of TB would be more cost-effective than testing only smear-positives [57]. In the present study Xpert MTB/RIF was used to reflexively test AFB smear-positive specimens, as well as AFB smear-negative specimens in patients with a high suspicion of pulmonary TB. While this study did not evaluate costs associated with its use, our results suggest that in a low incidence TB-setting the assay can be highly effective when applied under this protocol.

Limitations of the present study include its retrospective design, lack of clinical data, and relatively low numbers of RIF^*r*^ specimens. Information on treatment status was obtained for all patients with Xpert-positive culture-negative specimens, however it would be beneficial to incorporate further clinical data on all patients, allowing direct assessment of the assay’s impact on diagnostic and therapeutic decision-making. It would also be useful to evaluate the effect of Xpert MTB/RIF testing on other TB control efforts, such as contact tracing and respiratory isolation precautions, as well as the associated financial costs and benefits. Research in these areas will help to inform future clinical and programmatic use of the assay, and have the potential to impact broad public health strategies for TB control.

In summary, our results demonstrate that in a low TB-incidence setting and under routine practice conditions the Xpert MTB/RIF assay is able to accurately detect MTBC and RIF resistance in clinical sputum specimens, with performance equal to that seen in high-incidence settings. It provides the ability to rapidly diagnose or exclude pulmonary TB disease, differentiate MTBC from NTM, and detect suspected MDR TB; with markedly improved case detection over smear microscopy, particularly in low TB-incidence settings with high NTM-burden. Additionally, this study suggests that in a low TB-incidence setting, Xpert MTB/RIF may be effectively employed within a protocol for testing smear-positive and high-suspicion smear-negative sputum specimens.

## Supporting information

S1 File Minimal dataset. (PDF)

## References

[1] World Health Organization (WHO). Global Tuberculosis Report 2016. WHO/HTM/TB/2016.13. Geneva; 2016.

[2] World Health Organization (WHO)—Stop TB Partnership. The Global Plan to End TB 2016–2020: The Paradigm Shift. Geneva; 2015.

[3] Centers for Disease Control and Prevention (CDC). Controlling tuberculosis in the United States: recommendations from the American Thoracic Society, CDC, and the Infectious Diseases Society of America. MMWR Morb Mortal Wkly Rep. 2005;54: 1–81. 16267499

[4] Steingart KR, Ng V, Henry M, Hopewell PC, Ramsay A, Cunningham J, et al Sputum processing methods to improve the sensitivity of smear microscopy for tuberculosis: a systematic review. Lancet Infect Dis. 2006;6: 664–74. doi: 10.1016/S1473-3099(06)70602-8 17008175 10.1016/S1473-3099(06)70602-8

[5] Steingart KR, Henry M, Ng V, Hopewell PC, Ramsay A, Cunningham J, et al Fluorescence versus conventional sputum smear microscopy for tuberculosis: a systematic review. Lancet Infect Dis. 2006;6: 570–81. doi: 10.1016/S1473-3099(06)70578-3 16931408 10.1016/S1473-3099(06)70578-3

[6] American Thoracic Society/Centers for Disease Control and Prevention. Diagnostic standards and classification of tuberculosis in adults and children. Am J Respir Crit Care Med. 2000;161: 1376–95. doi: 10.1164/ajrccm.161.4.16141 10764337 10.1164/ajrccm.161.4.16141

[7] World Health Organization (WHO). Implementing tuberculosis diagnostics: A policy framework. WHO/HTM/TB/2015.11. Geneva; 2015.

[8] World Health Organization (WHO). Global Tuberculosis Report 2013. WHO/HTM/TB/2013.11. Geneva; 2013.

[9] Global Laboratory Initiative (GLI). Guide for providing technical support to TB laboratories in low- and middle-income countries. Geneva; 2015. http://www.stoptb.org/wg/gli/assets/documents/guideforprovidingtechnicalsupport_gb_web.pdf

[10] Global Laboratory Initiative (GLI). Mycobacteriology Laboratory Manual. Geneva; 2014. http://www.stoptb.org/wg/gli/assets/documents/gli_mycobacteriology_lab_manual_web.pdf

[11] Van Deun A, Aung KJM, Bola V, Lebeke R, Hossain MA, de Rijk WB, et al Rifampin drug resistance tests for tuberculosis: challenging the gold standard. J Clin Microbiol. 2013;51: 2633–40. doi: 10.1128/JCM.00553-13 23761144 10.1128/JCM.00553-13 PMC3719626

[12] Van Deun A, Barrera L, Bastian I, Fattorini L, Hoffmann H, Kam KM, et al Mycobacterium tuberculosis strains with highly discordant rifampin susceptibility test results. J Clin Microbiol. 2009;47: 3501–6. doi: 10.1128/JCM.01209-09 19759221 10.1128/JCM.01209-09 PMC2772627

[13] Rigouts L, Gumusboga M, De Rijk WB, Nduwamahoro E, Uwizeye C, De Jong B, et al Rifampin resistance missed in automated liquid culture system for Mycobacterium tuberculosis isolates with specific rpoB mutations. J Clin Microbiol. 2013;51: 2641–2645. doi: 10.1128/JCM.02741-12 23761146 10.1128/JCM.02741-12 PMC3719602

[14] Lin S-YG, Desmond EP. Molecular Diagnosis of Tuberculosis and Drug Resistance. Clin Lab Med. 2014;34: 297–314. doi: 10.1016/j.cll.2014.02.005 24856529 10.1016/j.cll.2014.02.005

[15] World Health Organization (WHO). Automated Real-Time Nucleic Acid Amplification Technology for Rapid and Simultaneous Detection of Tuberculosis and Rifampicin Resistance: Xpert MTB/RIF Assay for the Diagnosis of Pulmonary and Extrapulmonary TB in Adults and Children Policy Update. WHO/HT Geneva; 2013. 25473701

[16] Centers for Disease Control and Prevention (CDC). Updated Guidelines for the Use of Nucleic Acid Amplification Tests in the Diagnosis of Tuberculosis. MMWR Morb Mortal Wkly Rep. 2009;58: 7–10. 19145221

[17] Centers for Disease Control and Prevention (CDC). Report of Expert Consultations on Rapid Molecular Testing to Detect Drug-Resistant Tuberculosis in the United States. Atlanta; 2008. http://www.cdc.gov/tb/topic/laboratory/rapidmoleculartesting/moldstreport.pdf

[18] Moore DF, Guzman JA, Mikhail LT. Reduction in turnaround time for laboratory diagnosis of pulmonary tuberculosis by routine use of a nucleic acid amplification test. Diagn Microbiol Infect Dis. 2005;52: 247–54. doi: 10.1016/j.diagmicrobio.2005.02.014 15893903 10.1016/j.diagmicrobio.2005.02.014

[19] Dinnes J, Deeks J, Kunst H, Gibson A, Cummins E, Waugh N, et al A systematic review of rapid diagnostic tests for the detection of tuberculosis infection. Health Technol Assess. 2007;11: 1–196. 10.3310/hta11030 17266837

[20] World Health Organization (WHO). Molecular line probe assays for rapid screening of patients at risk of multidrug-resistant tuberculosis (MDR-TB) Policy Statement. Geneva; 2008.

[21] World Health Organization (WHO). Automated Real-time Nucleic Acid Amplification Technology for Rapid and Simultaneous Detection of Tuberculosis and Rifampicin Resistance: Xpert MTB/RIF System Policy Statement. WHO/HTM/TB/2011.4. Geneva; 2011. 26158191

[22] US Food and Drug Administration (FDA). 510(k) decision summary: de novo request for evaluation of automatic class III designation for the Xpert^®^ MTB/RIF Assay for use with the GeneXpert^®^ Instrument Systems, including the GeneXpert^®^ Diagnostic (Dx) Systems and the GeneXpert^®^ Infinity Systems. Silver Spring; 2013.

[23] US Food and Drug Administration (FDA). FDA permits marketing of first US test labeled for simultaneous detection of tuberculosis bacteria and resistance to the antibiotic rifampin. Silver Spring; 2013. 24195114

[24] Helb D, Jones M, Story E, Boehme C, Wallace E, Ho K, et al Rapid detection of Mycobacterium tuberculosis and rifampin resistance by use of on-demand, near-patient technology. J Clin Microbiol. 2010;48: 229–37. doi: 10.1128/JCM.01463-09 19864480 10.1128/JCM.01463-09 PMC2812290

[25] Pai M, Schito M. Tuberculosis diagnostics in 2015: landscape, priorities, needs, and prospects. J Infect Dis. 2015;211 Suppl: S21–8. 25765103 10.1093/infdis/jiu803 PMC4366576

[26] World Health Organization (WHO). Xpert MTB/RIF implementation manual: Technical and operational “how-to” practical considerations. WHO/HTM/TB/2014.1. Geneva; 2014. 25473699

[27] Cepheid. Xpert^®^ MTB/RIF assay—package insert. Sunnyvale, CA; 2013.

[28] Boehme CC, Nabeta P, Hillemann D, Nicol MP, Shenai S, Krapp F, et al Rapid Molecular Detection of Tuberculosis and Rifampin Resistance. N Engl J Med. 2010;363: 1005–1015. doi: 10.1056/NEJMoa0907847 20825313 10.1056/NEJMoa0907847 PMC2947799

[29] Boehme CC, Nicol MP, Nabeta P, Michael JS, Gotuzzo E, Tahirli R, et al Feasibility, diagnostic accuracy, and effectiveness of decentralised use of the Xpert MTB/RIF test for diagnosis of tuberculosis and multidrug resistance: a multicentre implementation study. Lancet. 2011;377: 1495–1505. doi: 10.1016/S0140-6736(11)60438-8 21507477 10.1016/S0140-6736(11)60438-8 PMC3085933

[30] Lawn SD, Nicol MP. Xpert^®^ MTB/RIF assay: development, evaluation and implementation of a new rapid molecular diagnostic for tuberculosis and rifampicin resistance. Future Microbiol. 2011;6: 1067–1082. doi: 10.2217/fmb.11.84 21958145 10.2217/fmb.11.84 PMC3252681

[31] Chang K, Lu W, Wang J, Zhang K, Jia S, Li F, et al Rapid and effective diagnosis of tuberculosis and rifampicin resistance with Xpert MTB/RIF assay: a meta-analysis. J Infect. 2012;64: 580–8. doi: 10.1016/j.jinf.2012.02.012 22381459 10.1016/j.jinf.2012.02.012

[32] Steingart KR, Schiller I, Horne DJ, Pai M, Boehme CC, Dendukuri N. Xpert MTB/RIF assay for pulmonary tuberculosis and rifampicin resistance in adults. Cochrane Database Syst Rev. 2014; CD009593 doi: 10.1002/14651858.CD009593.pub3 24448973 10.1002/14651858.CD009593.pub3 PMC4470349

[33] World Health Organization (WHO). Towards TB elimination: an action framework for low-incidence countries. WHO/HTM/TB/2014.13. Geneva; 2014.

[34] Bowles EC, Freyee B, Van Ingen J, Mulder B, Boeree MJ, Van Soolingen D. Xpert MTB/RIF, a novel automated polymerase chain reaction-based tool for the diagnosis of tuberculosis. Int J Tuberc Lung Dis. 2011;15: 988–989. doi: 10.5588/ijtld.10.0574 21682978 10.5588/ijtld.10.0574

[35] Deggim V, Somoskovi A, Voit A, Böttger EC, Bloemberg G V. Integrating the Xpert MTB/RIF assay into a diagnostic workflow for rapid detection of Mycobacterium tuberculosis in a low-prevalence area. J Clin Microbiol. 2013;51: 2396–2399. doi: 10.1128/JCM.00151-13 23616455 10.1128/JCM.00151-13 PMC3697699

[36] Ioannidis P, Papaventsis D, Karabela S, Nikolaou S, Panagi M, Raftopoulou E, et al Cepheid GeneXpert MTB/RIF assay for Mycobacterium tuberculosis detection and rifampin resistance identification in patients with substantial clinical indications of tuberculosis and smear-negative microscopy results. J Clin Microbiol. 2011;49: 3068–70. doi: 10.1128/JCM.00718-11 21677069 10.1128/JCM.00718-11 PMC3147726

[37] Malbruny B, Le Marrec G, Courageux K, Leclercq R, Cattoir V. Rapid and efficient detection of Mycobacterium tuberculosis in respiratory and non-respiratory samples [Technical note]. Int J Tuberc Lung Dis. 2011;15: 553–555. doi: 10.5588/ijtld.10.0497 21396219 10.5588/ijtld.10.0497

[38] Marlowe EM, Novak-Weekley SM, Cumpio J, Sharp SE, Momeny M a., Babst A, et al Evaluation of the cepheid xpert MTB/RIF assay for direct detection of mycobacterium tuberculosis complex in respiratory specimens. J Clin Microbiol. 2011;49: 1621–1623. doi: 10.1128/JCM.02214-10 21289151 10.1128/JCM.02214-10 PMC3122817

[39] Miller MB, Popowitch EB, Backlund MG, Ager EPC. Performance of Xpert MTB/RIF RUO assay and IS6110 real-time PCR for mycobacterium tuberculosis detection in clinical samples. J Clin Microbiol. 2011;49: 3458–3462. doi: 10.1128/JCM.05212-11 21849695 10.1128/JCM.05212-11 PMC3187315

[40] Williamson D a., Basu I, Bower J, Freeman JT, Henderson G, Roberts S a. An evaluation of the Xpert MTB/RIF assay and detection of false-positive rifampicin resistance in Mycobacterium tuberculosis. Diagn Microbiol Infect Dis. 2012;74: 207–209. doi: 10.1016/j.diagmicrobio.2012.06.013 22819240 10.1016/j.diagmicrobio.2012.06.013

[41] Sohn H, Aero AD, Menzies D, Behr M, Schwartzman K, Alvarez GG, et al Xpert MTB/RIF Testing in a Low Tuberculosis Incidence, High-Resource Setting: Limitations in Accuracy and Clinical Impact. Clin Infect Dis. 2014;58: 970–976. doi: 10.1093/cid/ciu022 24429440 10.1093/cid/ciu022

[42] County of San Diego Tuberculosis Control Program. Comparison of National, State and County Data for Tuberculosis, 2008–2015. 2016. http://www.sandiegocounty.gov/content/dam/sdc/hhsa/programs/phs/tuberculosis_control_program/Factshttables2015.

[43] County of San Diego Tuberculosis Control Program. 2014 Fact Sheet. 2015. http://www.sandiegocounty.gov/content/dam/sdc/hhsa/programs/phs/tuberculosis_control_program/Factsheet2014.pdf

[44] County of San Diego Tuberculosis Control Program. Tuberculosis Control Program 2013 Annual Report. San Diego; 2014. http://www.sandiegocounty.gov/content/dam/sdc/hhsa/programs/phs/documents/TBControl_AnnualReport2013.pdf

[45] Becton Dickinson. BACTEC MGIT 960 SIRE kit—package insert. Sparks, MD; 2015.

[46] Becton Dickinson. BACTEC MGIT 960 PZA kit—package insert. Sparks, MD; 2009.

[47] Lin S-YG, Rodwell TC, Victor TC, Rider EC, Pham L, Catanzaro A, et al Pyrosequencing for Rapid Detection of Extensively Drug-Resistant Mycobacterium tuberculosis in Clinical Isolates and Clinical Specimens. J Clin Microbiol. 2014;52: 475–482. doi: 10.1128/JCM.01821-13 24478476 10.1128/JCM.01821-13 PMC3911348

[48] Rodwell TC, Valafar F, Douglas J, Qian L, Garfein RS, Chawla A, et al Predicting Extensively Drug-Resistant Mycobacterium tuberculosis Phenotypes with Genetic Mutations. J Clin Microbiol. 2014;52: 781–789. doi: 10.1128/JCM.02701-13 24353002 10.1128/JCM.02701-13 PMC3957771

[49] Centers for Disease Control and Prevention (CDC). Availability of an assay for detecting Mycobacterium tuberculosis, including rifampin-resistant strains, and considerations for its use—United States, 2013. MMWR Morb Mortal Wkly Rep. 2013;62: 821–7. 24141407 PMC4585342

[50] Centers for Disease Control and Prevention (CDC). Report of an Expert Consultation on the Uses of Nucleic Acid Amplification Tests for the Diagnosis of Tuberculosis. Atlanta; 2008. http://www.cdc.gov/tb/publications/guidelines/amplification_test/amplification_tests.pdf

[51] Guerra RL, Hooper NM, Baker JF, Alborz R, Armstrong DT, Maltas G, et al Use of the amplified mycobacterium tuberculosis direct test in a public health laboratory: test performance and impact on clinical care. Chest. 2007;132: 946–51. doi: 10.1378/chest.06-2959 17573496 10.1378/chest.06-2959

[52] Mirsaeidi M, Machado RF, Garcia JGN, Schraufnagel DE. Nontuberculous mycobacterial disease mortality in the United States, 1999–2010: a population-based comparative study. PLoS One. 2014;9: e91879 doi: 10.1371/journal.pone.0091879 24632814 10.1371/journal.pone.0091879 PMC3954860

[53] McShane PJ, Glassroth J. Pulmonary Disease Due to Nontuberculous Mycobacteria. Chest. 2015;148: 1517–1527. doi: 10.1378/chest.15-0458 26225805 10.1378/chest.15-0458 PMC4665741

[54] Alonso M, Palacios JJ, Herranz M, Penedo A, Menéndez A, Bouza E, et al Isolation of Mycobacterium tuberculosis strains with a silent mutation in rpoB leading to potential misassignment of resistance category. J Clin Microbiol. 2011;49: 2688–90. doi: 10.1128/JCM.00659-11 21562104 10.1128/JCM.00659-11 PMC3147855

[55] McAlister AJ, Driscoll J, Metchock B. DNA sequencing for confirmation of rifampin resistance detected by cepheid xpert MTB/RIF assay. J Clin Microbiol. 2015;53: 1752–1753. doi: 10.1128/JCM.03433-14 25740782 10.1128/JCM.03433-14 PMC4400788

[56] LoBue PA, Moser KS. Isoniazid- and rifampin-resistant tuberculosis in San Diego County, California, United States, 1993–2002. Int J Tuberc Lung Dis. 2005;9: 501–506. 15875920

[57] Choi HW, Miele K, Dowdy D, Shah M. Cost-effectiveness of Xpert^®^ MTB/RIF for diagnosing pulmonary tuberculosis in the United States. Int J Tuberc Lung Dis. 2013;17: 1328–1335. doi: 10.5588/ijtld.13.0095 24025386 10.5588/ijtld.13.0095 PMC3891798

