# Supplementary material for: Performance of the Xpert MTB/RIF assay for the diagnosis of pulmonary tuberculosis and rifampin resistance in a low-incidence, high-resource setting
Source: PLoS One. 2017 Oct 9;12(10):e0186139. doi: 10.1371/journal.pone.0186139 (PMC5633176; doi:10.1371/journal.pone.0186139)
Supplement: S1 File — (PDF) [file pone.0186139.s001.pdf]

| Sp # | Pt # | Age | Xp | Xp2 | Xp3 | Sm | Sm2 | Cx | Cx2 | Cx3 | Eth | INH 0.1 | INH 0.4 | PZA | Rif | Rif(rev) | PSQ | On Tx | Coll |
|---|---|---|---|---|---|---|---|---|---|---|---|---|---|---|---|---|---|---|---|
| 1 | 1 | 51 | MTB - | MTB - | | 1+ | Pos | Neg | Neg | MTBC - | | | | | | | | | ES |
| 2 | 2 | 43 | MTB - | MTB - | | Neg | Neg | Neg | Neg | MTBC - | | | | | | | | | IS |
| 3 | 2 | 43 | MTB - | MTB - | | Neg | Neg | Neg | Neg | MTBC - | | | | | | | | | IS |
| 4 | 3 | 31 | MTB + / RIF - | MTB + | RIF - | 2+ | Pos | Neg | Neg | MTBC - | | | | | | | | Yes | IS |
| 5 | 4 | 50 | MTB - | MTB - | | 1+ | Pos | Neg | Neg | MTBC - | | | | | | | | | ES |
| 6 | 5 | 64 | MTB - | MTB - | | Neg | Neg | Neg | Neg | MTBC - | | | | | | | | | IS |
| 7 | 6 | 39 | MTB + / RIF + | MTB + | RIF + | Neg | Neg | MTB + | Pos | MTBC + | S | R | R | S | S | R | Disputed mutation | | IS |
| 8 | 7 | 60 | MTB - | MTB - | | Neg | Neg | MTB + | Pos | MTBC + | S | S | S | S | S | S | | | IS |
| 9 | 7 | 60 | MTB + / RIF - | MTB + | RIF - | 1+ | Pos | MTB + | Pos | MTBC + | S | S | S | S | S | S | | | ES |
| 10 | 8 | 36 | MTB - | MTB - | | Neg | Neg | NTM - MAC | NTM | MTBC - | | | | | | | | | IS |
| 11 | 9 | 60 | MTB - | MTB - | | Neg | Neg | Neg | Neg | MTBC - | | | | | | | | | IS |
| 12 | 10 | 35 | MTB - | MTB - | | Neg | Neg | Neg | Neg | MTBC - | | | | | | | | | IS |
| 13 | 11 | 43 | MTB - | MTB - | | Neg | Neg | NTM - MAC | NTM | MTBC - | | | | | | | | | IS |
| 14 | 11 | 46 | MTB - | MTB - | | Neg | Neg | Neg | Neg | MTBC - | | | | | | | | | IS |
| 15 | 12 | 60 | MTB - | MTB - | | Neg | Neg | NTM - M.gordonae | NTM | MTBC - | | | | | | | | | IS |
| 16 | 13 | 55 | MTB - | MTB - | | 1+ | Pos | NTM - M.gordonae | NTM | MTBC - | | | | | | | | | IS |
| 17 | 14 | 58 | MTB - | MTB - | | 2+ | Pos | NTM - MAC | NTM | MTBC - | | | | | | | | | IS |
| 18 | 14 | 58 | MTB - | MTB - | | 3+ | Pos | NTM - MAC | NTM | MTBC - | | | | | | | | | IS |
| 19 | 15 | 57 | MTB + / RIF - | MTB + | RIF - | 4+ | Pos | M.bovis | Pos | MTBC + | S | S | S | R | S | S | | | IS |
| 20 | 16 | 62 | MTB - | MTB - | | Neg | Neg | NTM - other M.species | NTM | MTBC - | | | | | | | | | IS |
| 21 | 17 | 57 | MTB - | MTB - | | Neg | Neg | NTM - M.gordonae | NTM | MTBC - | | | | | | | | | IS |
| 22 | 18 | 27 | MTB - | MTB - | | Neg | Neg | Neg | Neg | MTBC - | | | | | | | | | IS |
| 23 | 19 | 46 | MTB - | MTB - | | 1+ | Pos | Neg | Neg | MTBC - | | | | | | | | | IS |
| 24 | 20 | 38 | MTB - | MTB - | | 1+ | Pos | Neg | Neg | MTBC - | | | | | | | | | IS |
| 25 | 21 | 51 | MTB - | MTB - | | Neg | Neg | Neg | Neg | MTBC - | | | | | | | | | IS |
| 26 | 22 | 38 | MTB - | MTB - | | Neg | Neg | NTM - M.gordonae | NTM | MTBC - | | | | | | | | | IS |
| 27 | 23 | 39 | MTB - | MTB - | | Neg | Neg | NTM - rapid grower | NTM | MTBC - | | | | | | | | | IS |
| 28 | 24 | 54 | MTB + / RIF - | MTB + | RIF - | 1+ | Pos | MTB + | Pos | MTBC + | S | S | S | S | S | S | | | IS |
| 29 | 25 | 59 | MTB - | MTB - | | Neg | Neg | NTM - rapid grower | NTM | MTBC - | | | | | | | | | IS |
| 30 | 26 | 19 | MTB - | MTB - | | Neg | Neg | NTM - rapid grower | NTM | MTBC - | | | | | | | | | IS |
| 31 | 26 | 19 | MTB - | MTB - | | Neg | Neg | NTM - M.gordonae | NTM | MTBC - | | | | | | | | | IS |
| 32 | 27 | 40 | MTB + / RIF - | MTB + | RIF - | 1+ | Pos | Neg | Neg | MTBC - | | | | | | | | Yes | IS |
| 33 | 28 | 47 | MTB - | MTB - | | Neg | Neg | NTM - M.gordonae | NTM | MTBC - | | | | | | | | | IS |
| 34 | 29 | 69 | MTB - | MTB - | | Neg | Neg | NTM - other M.species | NTM | MTBC - | | | | | | | | | IS |
| 35 | 30 | 49 | MTB - | MTB - | | Neg | Neg | NTM - other M.species | NTM | MTBC - | | | | | | | | | ES |
| 36 | 31 | 14 | MTB + / RIF + | MTB + | RIF + | 4+ | Pos | MTB + | Pos | MTBC + | R | R | R | R | R | R | 531TTG mutation [illegible] | | IS |
| 37 | 32 | 17 | MTB + / RIF - | MTB + | RIF - | 2+ | Pos | MTB + | Pos | MTBC + | S | S | S | S | S | S | | | IS |
| 38 | 33 | 65 | MTB - | MTB - | | Neg | Neg | NTM - rapid grower | NTM | MTBC - | | | | | | | | | IS |
| 39 | 34 | 68 | MTB - | MTB - | | 1+ | Pos | NTM - M.gordonae | NTM | MTBC - | | | | | | | | | ES |
| 40 | 35 | 28 | MTB + / RIF - | MTB + | RIF - | 3+ | Pos | MTB + | Pos | MTBC + | S | S | S | S | S | S | | | IS |
| 41 | 36 | 62 | MTB + / RIF - | MTB + | RIF - | 1+ | Pos | MTB + | Pos | MTBC + | S | S | S | S | S | S | | | IS |
| 42 | 37 | 54 | MTB - | MTB - | | Neg | Neg | Neg | Neg | MTBC - | | | | | | | | | IS |

| | | | | | | | | | | | | | | | | | | | |
|---|---|---|---|---|---|---|---|---|---|---|---|---|---|---|---|---|---|---|---|
| 43 | 38 | 41 | MTB - | MTB - | | Neg | Neg | NTM - M.gordonae | NTM | MTBC - | | | | | | | | | IS |
| 44 | 39 | 54 | MTB + / RIF - | MTB + | RIF - | Neg | Neg | MTB + | Pos | MTBC + | S | S | S | S | S | S | | | IS |
| 45 | 40 | 62 | MTB - | MTB - | | Neg | Neg | NTM - other M.species | NTM | MTBC - | | | | | | | | | IS |
| 46 | 41 | 31 | MTB - | MTB - | | Neg | Neg | Neg | Neg | MTBC - | | | | | | | | | IS |
| 47 | 42 | 38 | MTB - | MTB - | | Neg | Neg | Neg | Neg | MTBC - | | | | | | | | | IS |
| 48 | 43 | 71 | MTB - | MTB - | | Neg | Neg | NTM - M.gordonae | NTM | MTBC - | | | | | | | | | IS |
| 49 | 44 | 34 | MTB + / RIF - | MTB + | RIF - | 1+ | Pos | MTB + | Pos | MTBC + | S | R | R | S | S | S | | | IS |
| 50 | 45 | 52 | MTB + / RIF - | MTB + | RIF - | 4+ | Pos | MTB + | Pos | MTBC + | S | S | S | S | S | S | | | ES |
| 51 | 46 | 29 | MTB + / RIF - | MTB + | RIF - | Neg | Neg | MTB + | Pos | MTBC + | S | S | S | S | S | S | | | IS |
| 52 | 47 | 42 | MTB - | MTB - | | Neg | Neg | Neg | Neg | MTBC - | | | | | | | | | IS |
| 53 | 48 | 29 | MTB + / RIF - | MTB + | RIF - | Neg | Neg | MTB + | Pos | MTBC + | S | S | S | S | S | S | | | ES |
| 54 | 49 | 24 | MTB + / RIF - | MTB + | RIF - | Neg | Neg | MTB + | Pos | MTBC + | S | S | S | S | S | S | | | IS |
| 55 | 50 | 17 | MTB - | MTB - | | Neg | Neg | Neg | Neg | MTBC - | | | | | | | | | IS |
| 56 | 50 | 17 | MTB - | MTB - | | Neg | Neg | Neg | Neg | MTBC - | | | | | | | | | ES |
| 57 | 51 | 29 | MTB - | MTB - | | Neg | Neg | Neg | Neg | MTBC - | | | | | | | | | IS |
| 58 | 52 | 49 | MTB - | MTB - | | Neg | Neg | Neg | Neg | MTBC - | | | | | | | | | ES |
| 59 | 53 | 53 | MTB - | MTB - | | Neg | Neg | Neg | Neg | MTBC - | | | | | | | | | ES |
| 60 | 54 | 32 | MTB - | MTB - | | Neg | Neg | Neg | Neg | MTBC - | | | | | | | | | ES |
| 61 | 55 | 20 | MTB + / RIF - | MTB + | RIF - | Neg | Neg | MTB + | Pos | MTBC + | S | S | S | S | S | S | | | IS |
| 62 | 56 | 45 | MTB - | MTB - | | Neg | Neg | Neg | Neg | MTBC - | | | | | | | | | ES |
| 63 | 57 | 74 | MTB + / RIF - | MTB + | RIF - | 3+ | Pos | MTB + | Pos | MTBC + | S | S | S | S | S | S | | | IS |
| 64 | 59 | 59 | MTB + / RIF - | MTB + | RIF - | Neg | Neg | MTB + | Pos | MTBC + | S | S | S | S | S | S | | | IS |
| 65 | 60 | 51 | MTB + / RIF - | MTB + | RIF - | Neg | Neg | MTB + | Pos | MTBC + | S | S | S | S | S | S | | | IS |
| 66 | 61 | 39 | MTB - | MTB - | | Neg | Neg | Neg | Neg | MTBC - | | | | | | | | | ES |
| 67 | 62 | 13 | MTB + / RIF - | MTB + | RIF - | 3+ | Pos | MTB + | Pos | MTBC + | S | R | R | S | S | S | | | IS |
| 68 | 62 | 13 | MTB + / RIF - | MTB + | RIF - | 1+ | Pos | Neg | Neg | MTBC - | | | | | | | | Yes | IS |
| 69 | 63 | 87 | MTB + / RIF - | MTB + | RIF - | 1+ | Pos | MTB + | Pos | MTBC + | S | S | S | S | S | S | | | IS |
| 70 | 64 | 55 | MTB + / RIF - | MTB + | RIF - | Neg | Neg | MTB + | Pos | MTBC + | S | S | S | S | S | S | | | IS |
| 71 | 65 | 42 | MTB - | MTB - | | 2+ | Pos | Neg | Neg | MTBC - | | | | | | | | | IS |
| 72 | 66 | 42 | MTB - | MTB - | | 2+ | Pos | Neg | Neg | MTBC - | | | | | | | | | ES |
| 73 | 67 | 65 | MTB + / RIF - | MTB + | RIF - | 1+ | Pos | Neg | Neg | MTBC - | | | | | | | | Yes | IS |
| 74 | 68 | 48 | MTB + / RIF - | MTB + | RIF - | 4+ | Pos | MTB + | Pos | MTBC + | S | S | S | S | S | S | | | IS |
| 75 | 69 | 54 | MTB - | MTB - | | Neg | Neg | Neg | Neg | MTBC - | | | | | | | | | ES |
| 76 | 70 | 19 | MTB - | MTB - | | Neg | Neg | Neg | Neg | MTBC - | | | | | | | | | ES |
| 77 | 71 | 43 | MTB - | MTB - | | Neg | Neg | Neg | Neg | MTBC - | | | | | | | | | IS |
| 78 | 72 | 22 | MTB - | MTB - | | Neg | Neg | Neg | Neg | MTBC - | | | | | | | | | ES |
| 79 | 73 | 35 | MTB - | MTB - | | Neg | Neg | Neg | Neg | MTBC - | | | | | | | | | IS |
| 80 | 74 | 35 | MTB + / RIF - | MTB + | RIF - | 2+ | Pos | MTB + | Pos | MTBC + | S | S | S | S | S | S | | | IS |
| 81 | 75 | 22 | MTB + / RIF - | MTB + | RIF - | 3+ | Pos | MTB + | Pos | MTBC + | S | S | S | S | S | S | | | IS |
| 82 | 76 | 20 | MTB + / RIF - | MTB + | RIF - | 4+ | Pos | MTB + | Pos | MTBC + | S | S | S | S | S | S | | | IS |
| 83 | 77 | 89 | MTB + / RIF - | MTB + | RIF - | Neg | Neg | MTB + | Pos | MTBC + | S | S | S | S | S | S | | | IS |
| 84 | 78 | 31 | MTB + / RIF - | MTB + | RIF - | Neg | Neg | MTB + | Pos | MTBC + | S | S | S | S | S | S | | | IS |
| 85 | 80 | 60 | MTB - | MTB - | | Neg | Neg | Neg | Neg | MTBC - | | | | | | | | | IS |

| | | | | | | | | | | | | | | | | | | | |
|---|---|---|---|---|---|---|---|---|---|---|---|---|---|---|---|---|---|---|---|
| 86 | 81 | 60 | MTB - | MTB - | | Neg | Neg | Neg | Neg | MTBC - | | | | | | | | | IS |
| 87 | 82 | 72 | MTB + / RIF - | MTB + | RIF - | Neg | Neg | MTB + | Pos | MTBC + | S | S | S | S | S | S | | | IS |
| 88 | 83 | 27 | MTB + / RIF - | MTB + | RIF - | 4+ | Pos | MTB + | Pos | MTBC + | S | S | S | S | S | S | | | IS |
| 89 | 84 | 64 | MTB - | MTB - | | Neg | Neg | Neg | Neg | MTBC - | | | | | | | | | IS |
| 90 | 85 | 11 | MTB - | MTB - | | Neg | Neg | Neg | Neg | MTBC - | | | | | | | | | IS |
| 91 | 86 | 75 | MTB - | MTB - | | Neg | Neg | NTM - MAC | NTM | MTBC - | | | | | | | | | IS |
| 92 | 87 | 43 | MTB + / RIF - | MTB + | RIF - | Neg | Neg | MTB + | Pos | MTBC + | S | S | S | S | S | S | | | IS |
| 93 | 88 | 67 | MTB - | MTB - | | 1+ | Pos | Neg | Neg | MTBC - | | | | | | | | | ES |
| 94 | 89 | 42 | MTB - | MTB - | | Neg | Neg | Neg | Neg | MTBC - | | | | | | | | | IS |
| 95 | 90 | 50 | MTB - | MTB - | | 1+ | Pos | NTM - M.gordonae | NTM | MTBC - | | | | | | | | | IS |
| 96 | 91 | 65 | MTB - | MTB - | | Neg | Neg | Neg | Neg | MTBC - | | | | | | | | | IS |
| 97 | 92 | 71 | MTB + / RIF - | MTB + | RIF - | 1+ | Pos | Neg | Neg | MTBC - | | | | | | | | Yes | IS |
| 98 | 93 | 58 | MTB + / RIF - | MTB + | RIF - | Neg | Neg | MTB + | Pos | MTBC + | S | S | S | S | S | S | | | IS |
| 99 | 93 | 59 | MTB - | MTB - | | Neg | Neg | Neg | Neg | MTBC - | | | | | | | | | IS |
| 100 | 94 | 61 | MTB - | MTB - | | 2+ | Pos | Neg | Neg | MTBC - | | | | | | | | | IS |
| 101 | 95 | 38 | MTB - | MTB - | | Neg | Neg | Neg | Neg | MTBC - | | | | | | | | | IS |
| 102 | 96 | 53 | MTB - | MTB - | | Neg | Neg | Neg | Neg | MTBC - | | | | | | | | | IS |
| 103 | 96 | 53 | MTB - | MTB - | | Neg | Neg | Neg | Neg | MTBC - | | | | | | | | | IS |
| 104 | 97 | 44 | MTB - | MTB - | | Neg | Neg | Neg | Neg | MTBC - | | | | | | | | | IS |
| 105 | 98 | 77 | MTB - | MTB - | | Neg | Neg | Neg | Neg | MTBC - | | | | | | | | | IS |
| 106 | 99 | 28 | MTB - | MTB - | | 1+ | Pos | NTM - M.gordonae | NTM | MTBC - | | | | | | | | | IS |
| 107 | 100 | 48 | MTB - | MTB - | | 1+ | Pos | NTM - rapid grower | NTM | MTBC - | | | | | | | | | IS |
| 108 | 101 | 62 | MTB - | MTB - | | Neg | Neg | Neg | Neg | MTBC - | | | | | | | | | IS |
| 109 | 102 | 56 | MTB - | MTB - | | Neg | Neg | Neg | Neg | MTBC - | | | | | | | | | IS |
| 110 | 103 | 80 | MTB + / RIF - | MTB + | RIF - | 3+ | Pos | MTB + | Pos | MTBC + | S | S | S | S | S | S | | | IS |
| 111 | 104 | 62 | MTB - | MTB - | | Neg | Neg | Neg | Neg | MTBC - | | | | | | | | | IS |
| 112 | 105 | 24 | MTB - | MTB - | | Neg | Neg | Neg | Neg | MTBC - | | | | | | | | | IS |
| 113 | 106 | 56 | MTB - | MTB - | | Neg | Neg | Neg | Neg | MTBC - | | | | | | | | | IS |
| 114 | 107 | 31 | MTB - | MTB - | | Neg | Neg | Neg | Neg | MTBC - | | | | | | | | | IS |
| 115 | 108 | 57 | MTB - | MTB - | | 1+ | Pos | Neg | Neg | MTBC - | | | | | | | | | IS |
| 116 | 109 | 42 | MTB - | MTB - | | Neg | Neg | Neg | Neg | MTBC - | | | | | | | | | IS |
| 117 | 109 | 43 | MTB + / RIF - | MTB + | RIF - | Neg | Neg | MTB + | Pos | MTBC + | S | S | S | S | S | S | | | IS |
| 118 | 109 | 43 | MTB - | MTB - | | Neg | Neg | NTM - M.gordonae | NTM | MTBC - | | | | | | | | | IS |
| 119 | 110 | 27 | MTB - | MTB - | | Neg | Neg | Neg | Neg | MTBC - | | | | | | | | | IS |
| 120 | 111 | 42 | MTB - | MTB - | | Neg | Neg | Neg | Neg | MTBC - | | | | | | | | | IS |
| 121 | 111 | 42 | MTB - | MTB - | | Neg | Neg | Neg | Neg | MTBC - | | | | | | | | | IS |
| 122 | 112 | 53 | MTB - | MTB - | | Neg | Neg | Neg | Neg | MTBC - | | | | | | | | | IS |
| 123 | 113 | 62 | MTB + / RIF - | MTB + | RIF - | 1+ | Pos | MTB + | Pos | MTBC + | S | R | R | S | S | S | | | ES |
| 124 | 114 | 49 | MTB + / RIF - | MTB + | RIF - | 4+ | Pos | MTB + | Pos | MTBC + | S | S | S | S | S | S | | | IS |
| 125 | 115 | 57 | MTB - | MTB - | | Neg | Neg | Neg | Neg | MTBC - | | | | | | | | | IS |
| 126 | 116 | 51 | MTB + / RIF - | MTB + | RIF - | 2+ | Pos | MTB + | Pos | MTBC + | S | S | S | S | S | S | | | IS |
| 127 | 116 | 52 | MTB + / RIF - | MTB + | RIF - | 2+ | Pos | Neg | Neg | MTBC - | | | | | | | | Yes | IS |
| 128 | 117 | 37 | MTB + / RIF - | MTB + | RIF - | 2+ | Pos | MTB + | Pos | MTBC + | S | S | S | S | S | S | | | IS |

| | | | | | | | | | | | | | | | | | | | |
|---|---|---|---|---|---|---|---|---|---|---|---|---|---|---|---|---|---|---|---|
| 129 | 118 | 42 | MTB - | MTB - | | Neg | Neg | Neg | Neg | MTBC - | | | | | | | | | IS |
| 130 | 119 | 55 | MTB - | MTB - | | Neg | Neg | Neg | Neg | MTBC - | | | | | | | | | IS |
| 131 | 120 | 41 | MTB - | MTB - | | Neg | Neg | Neg | Neg | MTBC - | | | | | | | | | IS |
| 132 | 121 | 48 | MTB - | MTB - | | Neg | Neg | Neg | Neg | MTBC - | | | | | | | | | IS |
| 133 | 122 | 63 | MTB - | MTB - | | Neg | Neg | Neg | Neg | MTBC - | | | | | | | | | IS |
| 134 | 123 | 54 | MTB - | MTB - | | Neg | Neg | Neg | Neg | MTBC - | | | | | | | | | IS |
| 135 | 124 | 21 | MTB - | MTB - | | Neg | Neg | Neg | Neg | MTBC - | | | | | | | | | IS |
| 136 | 125 | 25 | MTB - | MTB - | | Neg | Neg | Neg | Neg | MTBC - | | | | | | | | | IS |
| 137 | 127 | 42 | MTB - | MTB - | | Neg | Neg | Neg | Neg | MTBC - | | | | | | | | | IS |
| 138 | 128 | 91 | MTB - | MTB - | | Neg | Neg | Neg | Neg | MTBC - | | | | | | | | | IS |
| 139 | 129 | 54 | MTB - | MTB - | | Neg | Neg | Neg | Neg | MTBC - | | | | | | | | | IS |
| 140 | 130 | 19 | MTB - | MTB - | | Neg | Neg | Neg | Neg | MTBC - | | | | | | | | | IS |
| 141 | 131 | 33 | MTB - | MTB - | | Neg | Neg | Neg | Neg | MTBC - | | | | | | | | | IS |
| 142 | 131 | 33 | MTB - | MTB - | | Neg | Neg | Neg | Neg | MTBC - | | | | | | | | | IS |
| 143 | 132 | 18 | MTB + / RIF - | MTB + | RIF - | 3+ | Pos | MTB + | Pos | MTBC + | S | S | S | S | S | S | | | IS |
| 144 | 133 | 69 | MTB - | MTB - | | Neg | Neg | Neg | Neg | MTBC - | | | | | | | | | IS |
| 145 | 133 | 69 | MTB - | MTB - | | Neg | Neg | Neg | Neg | MTBC - | | | | | | | | | IS |
| 146 | 134 | 60 | MTB - | MTB - | | Neg | Neg | Neg | Neg | MTBC - | | | | | | | | | IS |
| 147 | 135 | 52 | MTB - | MTB - | | 1+ | Pos | NTM - M.kansasii | NTM | MTBC - | | | | | | | | | IS |
| 148 | 136 | 72 | MTB - | MTB - | | Neg | Neg | Neg | Neg | MTBC - | | | | | | | | | IS |
| 149 | 137 | 57 | MTB + / RIF - | MTB + | RIF - | 4+ | Pos | MTB + | Pos | MTBC + | S | S | S | S | S | S | | | IS |
| 150 | 138 | 14 | MTB - | MTB - | | Neg | Neg | Neg | Neg | MTBC - | | | | | | | | | IS |
| 151 | 139 | 29 | MTB - | MTB - | | Neg | Neg | NTM - MAC | NTM | MTBC - | | | | | | | | | IS |
| 152 | 139 | 29 | MTB - | MTB - | | Neg | Neg | NTM - rapid grower | NTM | MTBC - | | | | | | | | | IS |
| 153 | 139 | 29 | MTB - | MTB - | | 1+ | Pos | NTM - rapid grower | NTM | MTBC - | | | | | | | | | IS |
| 154 | 140 | 57 | MTB - | MTB - | | Neg | Neg | NTM - MAC | NTM | MTBC - | | | | | | | | | IS |
| 155 | 140 | 57 | MTB - | MTB - | | Neg | Neg | NTM - MAC | NTM | MTBC - | | | | | | | | | IS |
| 156 | 141 | 65 | MTB - | MTB - | | Neg | Neg | Neg | Neg | MTBC - | | | | | | | | | IS |
| 157 | 142 | 22 | MTB - | MTB - | | Neg | Neg | Neg | Neg | MTBC - | | | | | | | | | IS |
| 158 | 143 | 41 | MTB - | MTB - | | Neg | Neg | Neg | Neg | MTBC - | | | | | | | | | IS |
| 159 | 144 | 48 | MTB - | MTB - | | Neg | Neg | Neg | Neg | MTBC - | | | | | | | | | IS |
| 160 | 145 | 61 | MTB - | MTB - | | Neg | Neg | Neg | Neg | MTBC - | | | | | | | | | IS |
| 161 | 146 | 59 | MTB - | MTB - | | Neg | Neg | Neg | Neg | MTBC - | | | | | | | | | IS |
| 162 | 147 | 58 | MTB - | MTB - | | Neg | Neg | Neg | Neg | MTBC - | | | | | | | | | IS |
| 163 | 148 | 45 | MTB - | MTB - | | Neg | Neg | Neg | Neg | MTBC - | | | | | | | | | IS |
| 164 | 149 | 21 | MTB + / RIF - | MTB + | RIF - | 4+ | Pos | MTB + | Pos | MTBC + | S | S | S | S | S | S | | | ES |
| 165 | 150 | 48 | MTB - | MTB - | | Neg | Neg | Neg | Neg | MTBC - | | | | | | | | | IS |
| 166 | 151 | 34 | MTB + / RIF - | MTB + | RIF - | 1+ | Pos | MTB + | Pos | MTBC + | S | S | S | S | S | S | | | IS |
| 167 | 152 | 30 | MTB - | MTB - | | Neg | Neg | Neg | Neg | MTBC - | | | | | | | | | ES |
| 168 | 153 | 57 | MTB - | MTB - | | Neg | Neg | Neg | Neg | MTBC - | | | | | | | | | IS |
| 169 | 154 | 80 | MTB - | MTB - | | Neg | Neg | Neg | Neg | MTBC - | | | | | | | | | IS |
| 170 | 155 | 43 | MTB - | MTB - | | Neg | Neg | Neg | Neg | MTBC - | | | | | | | | | IS |
| 171 | 156 | 41 | MTB - | MTB - | | Neg | Neg | Neg | Neg | MTBC - | | | | | | | | | IS |

| | | | | | | | | | | | | | | | | | | | |
|---|---|---|---|---|---|---|---|---|---|---|---|---|---|---|---|---|---|---|---|
| 172 | 157 | 58 | MTB - | MTB - | | 1+ | Pos | Neg | Neg | MTBC - | | | | | | | | | ES |
| 173 | 158 | 46 | MTB - | MTB - | | Neg | Neg | Neg | Neg | MTBC - | | | | | | | | | IS |
| 174 | 159 | 48 | MTB - | MTB - | | Neg | Neg | Neg | Neg | MTBC - | | | | | | | | | IS |
| 175 | 160 | 74 | MTB - | MTB - | | Neg | Neg | Neg | Neg | MTBC - | | | | | | | | | IS |
| 176 | 160 | 75 | MTB - | MTB - | | Neg | Neg | Neg | Neg | MTBC - | | | | | | | | | IS |
| 177 | 160 | 75 | MTB - | MTB - | | Neg | Neg | Neg | Neg | MTBC - | | | | | | | | | IS |
| 178 | 161 | 73 | MTB - | MTB - | | Neg | Neg | Neg | Neg | MTBC - | | | | | | | | | IS |
| 179 | 162 | 58 | MTB + / RIF - | MTB + | RIF - | 1+ | Pos | MTB + | Pos | MTBC + | S | S | S | S | S | S | | | IS |
| 180 | 163 | 67 | MTB + / RIF - | MTB + | RIF - | Neg | Neg | MTB + | Pos | MTBC + | S | R | S | S | S | S | | | IS |
| 181 | 164 | 61 | MTB - | MTB - | | Neg | Neg | Neg | Neg | MTBC - | | | | | | | | | IS |
| 182 | 165 | 45 | MTB - | MTB - | | Neg | Neg | Neg | Neg | MTBC - | | | | | | | | | IS |
| 183 | 166 | 53 | MTB - | MTB - | | Neg | Neg | Neg | Neg | MTBC - | | | | | | | | | IS |
| 184 | 166 | 53 | MTB - | MTB - | | 1+ | Pos | Neg | Neg | MTBC - | | | | | | | | | IS |
| 185 | 167 | 16 | MTB - | MTB - | | Neg | Neg | Neg | Neg | MTBC - | | | | | | | | | IS |
| 186 | 168 | 36 | MTB - | MTB - | | Neg | Neg | Neg | Neg | MTBC - | | | | | | | | | IS |
| 187 | 169 | 73 | MTB - | MTB - | | Neg | Neg | Neg | Neg | MTBC - | | | | | | | | | IS |
| 188 | 170 | 61 | MTB - | MTB - | | Neg | Neg | Neg | Neg | MTBC - | | | | | | | | | IS |
| 189 | 171 | 27 | MTB - | MTB - | | Neg | Neg | Neg | Neg | MTBC - | | | | | | | | | IS |
| 190 | 172 | 43 | MTB - | MTB - | | Neg | Neg | MTB + | Pos | MTBC + | S | S | S | S | S | S | | | IS |
| 191 | 172 | 43 | MTB + / RIF - | MTB + | RIF - | 2+ | Pos | MTB + | Pos | MTBC + | S | S | S | S | S | S | | | IS |
| 192 | 172 | 43 | MTB - | MTB - | | Neg | Neg | Neg | Neg | MTBC - | | | | | | | | | IS |
| 193 | 173 | 30 | MTB - | MTB - | | 1+ | Pos | NTM - M.gordonae | NTM | MTBC - | | | | | | | | | ES |
| 194 | 173 | 31 | MTB - | MTB - | | Neg | Neg | NTM - rapid grower | NTM | MTBC - | | | | | | | | | ES |
| 195 | 174 | 78 | MTB - | MTB - | | Neg | Neg | Neg | Neg | MTBC - | | | | | | | | | IS |
| 196 | 175 | 42 | MTB - | MTB - | | Neg | Neg | Neg | Neg | MTBC - | | | | | | | | | IS |
| 197 | 176 | 31 | MTB + / RIF - | MTB + | RIF - | 4+ | Pos | MTB + | Pos | MTBC + | S | S | S | S | S | S | | | IS |
| 198 | 177 | 34 | MTB - | MTB - | | Neg | Neg | Neg | Neg | MTBC - | | | | | | | | | IS |
| 199 | 178 | 11 | MTB - | MTB - | | Neg | Neg | Neg | Neg | MTBC - | | | | | | | | | IS |
| 200 | 179 | 76 | MTB - | MTB - | | Neg | Neg | Neg | Neg | MTBC - | | | | | | | | | IS |
| 201 | 180 | 44 | MTB - | MTB - | | Neg | Neg | Neg | Neg | MTBC - | | | | | | | | | IS |
| 202 | 181 | 45 | MTB - | MTB - | | Neg | Neg | Neg | Neg | MTBC - | | | | | | | | | IS |
| 203 | 182 | 43 | MTB - | MTB - | | Neg | Neg | Neg | Neg | MTBC - | | | | | | | | | IS |
| 204 | 183 | 69 | MTB - | MTB - | | Neg | Neg | Neg | Neg | MTBC - | | | | | | | | | IS |
| 205 | 183 | 70 | MTB - | MTB - | | 1+ | Pos | Neg | Neg | MTBC - | | | | | | | | | IS |
| 206 | 184 | 41 | MTB - | MTB - | | Neg | Neg | Neg | Neg | MTBC - | | | | | | | | | IS |
| 207 | 185 | 49 | MTB + / RIF - | MTB + | RIF - | 4+ | Pos | MTB + | Pos | MTBC + | S | S | S | S | S | S | | | ES |
| 208 | 186 | 57 | MTB - | MTB - | | Neg | Neg | Neg | Neg | MTBC - | | | | | | | | | IS |
| 209 | 186 | 57 | MTB - | MTB - | | 1+ | Pos | NTM - rapid grower | NTM | MTBC - | | | | | | | | | IS |
| 210 | 187 | 55 | MTB - | MTB - | | Neg | Neg | Neg | Neg | MTBC - | | | | | | | | | IS |
| 211 | 188 | 27 | MTB - | MTB - | | Neg | Neg | Neg | Neg | MTBC - | | | | | | | | | IS |
| 212 | 189 | 47 | MTB - | MTB - | | Neg | Neg | Neg | Neg | MTBC - | | | | | | | | | IS |
| 213 | 190 | 18 | MTB + / RIF - | MTB + | RIF - | 1+ | Pos | MTB + | Pos | MTBC + | S | S | S | S | S | S | | | IS |
| 214 | 191 | 69 | MTB - | MTB - | | Neg | Neg | NTM - M.gordonae | NTM | MTBC - | | | | | | | | | IS |

| | | | | | | | | | | | | | | | | | | | |
|---|---|---|---|---|---|---|---|---|---|---|---|---|---|---|---|---|---|---|---|
| 215 | 192 | 29 | MTB - | MTB - | | Neg | Neg | Neg | Neg | MTBC - | | | | | | | | | IS |
| 216 | 193 | 64 | MTB - | MTB - | | Neg | Neg | Neg | Neg | MTBC - | | | | | | | | | IS |
| 217 | 194 | 29 | MTB - | MTB - | | Neg | Neg | Neg | Neg | MTBC - | | | | | | | | | IS |
| 218 | 195 | 59 | MTB - | MTB - | | Neg | Neg | Neg | Neg | MTBC - | | | | | | | | | IS |
| 219 | 196 | 20 | MTB + / RIF - | MTB + | RIF - | Neg | Neg | MTB + | Pos | MTBC + | S | S | S | S | S | S | | | ES |
| 220 | 197 | 55 | MTB - | MTB - | | Neg | Neg | Neg | Neg | MTBC - | | | | | | | | | IS |
| 221 | 198 | 45 | MTB - | MTB - | | Neg | Neg | Neg | Neg | MTBC - | | | | | | | | | IS |
| 222 | 199 | 30 | MTB - | MTB - | | Neg | Neg | Neg | Neg | MTBC - | | | | | | | | | IS |
| 223 | 200 | 63 | MTB - | MTB - | | Neg | Neg | MTB + | Pos | MTBC + | S | R | S | S | S | S | | | IS |
| 224 | 200 | 64 | MTB - | MTB - | | Neg | Neg | NTM - M.gordonae | NTM | MTBC - | | | | | | | | | ES |
| 225 | 201 | 66 | MTB - | MTB - | | Neg | Neg | NTM - rapid grower | NTM | MTBC - | | | | | | | | | IS |
| 226 | 202 | 46 | MTB - | MTB - | | Neg | Neg | Neg | Neg | MTBC - | | | | | | | | | IS |
| 227 | 203 | 36 | MTB + / RIF - | MTB + | RIF - | Neg | Neg | MTB + | Pos | MTBC + | S | S | S | S | S | S | | | IS |
| 228 | 203 | 36 | MTB - | MTB - | | Neg | Neg | Neg | Neg | MTBC - | | | | | | | | | IS |
| 229 | 204 | 25 | MTB + / RIF - | MTB + | RIF - | Neg | Neg | MTB + | Pos | MTBC + | S | S | S | S | S | S | | | IS |
| 230 | 205 | 55 | MTB - | MTB - | | Neg | Neg | Neg | Neg | MTBC - | | | | | | | | | IS |
| 231 | 206 | 23 | MTB - | MTB - | | Neg | Neg | Neg | Neg | MTBC - | | | | | | | | | IS |
| 232 | 207 | 21 | MTB - | MTB - | | Neg | Neg | NTM - rapid grower | NTM | MTBC - | | | | | | | | | IS |
| 233 | 208 | 26 | MTB - | MTB - | | Neg | Neg | MTB + | Pos | MTBC + | S | S | S | S | S | S | | | IS |
| 234 | 209 | 23 | MTB - | MTB - | | Neg | Neg | Neg | Neg | MTBC - | | | | | | | | | IS |
| 235 | 210 | 53 | MTB - | MTB - | | Neg | Neg | NTM - rapid grower | NTM | MTBC - | | | | | | | | | IS |
| 236 | 211 | 69 | MTB - | MTB - | | Neg | Neg | Neg | Neg | MTBC - | | | | | | | | | IS |
| 237 | 212 | 44 | MTB - | MTB - | | Neg | Neg | Neg | Neg | MTBC - | | | | | | | | | IS |
| 238 | 212 | 45 | MTB - | MTB - | | 1+ | Pos | Neg | Neg | MTBC - | | | | | | | | | IS |
| 239 | 213 | 48 | MTB - | MTB - | | Neg | Neg | Neg | Neg | MTBC - | | | | | | | | | IS |
| 240 | 214 | 49 | MTB - | MTB - | | Neg | Neg | Neg | Neg | MTBC - | | | | | | | | | IS |
| 241 | 215 | 36 | MTB - | MTB - | | Neg | Neg | Neg | Neg | MTBC - | | | | | | | | | IS |
| 242 | 216 | 67 | MTB - | MTB - | | Neg | Neg | Neg | Neg | MTBC - | | | | | | | | | IS |
| 243 | 216 | 67 | MTB - | MTB - | | Neg | Neg | NTM - M.gordonae | NTM | MTBC - | | | | | | | | | IS |
| 244 | 217 | 53 | MTB - | MTB - | | Neg | Neg | Neg | Neg | MTBC - | | | | | | | | | IS |
| 245 | 218 | 10 | MTB - | MTB - | | Neg | Neg | Neg | Neg | MTBC - | | | | | | | | | IS |
| 246 | 219 | 30 | MTB - | MTB - | | Neg | Neg | Neg | Neg | MTBC - | | | | | | | | | IS |
| 247 | 220 | 57 | MTB - | MTB - | | Neg | Neg | Neg | Neg | MTBC - | | | | | | | | | IS |
| 248 | 220 | 59 | MTB - | MTB - | | Neg | Neg | Neg | Neg | MTBC - | | | | | | | | | IS |
| 249 | 221 | 25 | MTB + / RIF - | MTB + | RIF - | 2+ | Pos | MTB + | Pos | MTBC + | S | R | R | S | S | S | | | IS |
| 250 | 222 | 33 | MTB - | MTB - | | Neg | Neg | Neg | Neg | MTBC - | | | | | | | | | IS |
| 251 | 223 | 58 | MTB - | MTB - | | Neg | Neg | Neg | Neg | MTBC - | | | | | | | | | IS |
| 252 | 224 | 56 | MTB - | MTB - | | Neg | Neg | Neg | Neg | MTBC - | | | | | | | | | IS |
| 253 | 225 | 58 | MTB - | MTB - | | Neg | Neg | Neg | Neg | MTBC - | | | | | | | | | IS |
| 254 | 226 | 68 | MTB - | MTB - | | Neg | Neg | Neg | Neg | MTBC - | | | | | | | | | IS |
| 255 | 226 | 69 | MTB - | MTB - | | Neg | Neg | Neg | Neg | MTBC - | | | | | | | | | IS |
| 256 | 227 | 30 | MTB - | MTB - | | Neg | Neg | Neg | Neg | MTBC - | | | | | | | | | IS |
| 257 | 228 | 51 | MTB - | MTB - | | Neg | Neg | Neg | Neg | MTBC - | | | | | | | | | IS |

| | | | | | | | | | | | | | | | | | | | |
|---|---|---|---|---|---|---|---|---|---|---|---|---|---|---|---|---|---|---|---|
| 258 | 229 | 63 | MTB - | MTB - | | Neg | Neg | Neg | Neg | MTBC - | | | | | | | | | IS |
| 259 | 230 | 56 | MTB - | MTB - | | Neg | Neg | NTM - MAC | NTM | MTBC - | | | | | | | | | IS |
| 260 | 231 | 46 | MTB + / RIF - | MTB + | RIF - | Neg | Neg | MTB + | Pos | MTBC + | S | S | S | S | S | S | | | IS |
| 261 | 231 | 47 | MTB - | MTB - | | Neg | Neg | NTM - other M.species | NTM | MTBC - | | | | | | | | | ES |
| 262 | 231 | 47 | MTB + / RIF - | MTB + | RIF - | 1+ | Pos | Neg | Neg | MTBC - | | | | | | | | Yes | ES |
| 263 | 232 | 38 | MTB - | MTB - | | Neg | Neg | Neg | Neg | MTBC - | | | | | | | | | IS |
| 264 | 232 | 38 | MTB - | MTB - | | Neg | Neg | NTM - rapid grower | NTM | MTBC - | | | | | | | | | IS |
| 265 | 233 | 25 | MTB + / RIF - | MTB + | RIF - | 4+ | Pos | MTB + | Pos | MTBC + | S | S | S | S | S | S | | | IS |
| 266 | 233 | 26 | MTB - | MTB - | | 1+ | Pos | Neg | Neg | MTBC - | | | | | | | | | IS |
| 267 | 233 | 26 | MTB - | MTB - | | 2+ | Pos | NTM - M.gordonae | NTM | MTBC - | | | | | | | | | IS |
| 268 | 233 | 26 | MTB - | MTB - | | Neg | Neg | NTM - M.gordonae | NTM | MTBC - | | | | | | | | | IS |
| 269 | 233 | 26 | MTB - | MTB - | | Neg | Neg | Neg | Neg | MTBC - | | | | | | | | | IS |
| 270 | 234 | 51 | MTB - | MTB - | | Neg | Neg | Neg | Neg | MTBC - | | | | | | | | | IS |
| 271 | 235 | 52 | MTB - | MTB - | | Neg | Neg | Neg | Neg | MTBC - | | | | | | | | | IS |
| 272 | 236 | 81 | MTB - | MTB - | | Neg | Neg | NTM - M.gordonae | NTM | MTBC - | | | | | | | | | ES |
| 273 | 237 | 23 | MTB + / RIF - | MTB + | RIF - | 4+ | Pos | MTB + | Pos | MTBC + | S | S | S | S | S | S | | | IS |
| 274 | 238 | 28 | MTB - | MTB - | | Neg | Neg | Neg | Neg | MTBC - | | | | | | | | | IS |
| 275 | 239 | 56 | MTB - | MTB - | | Neg | Neg | Neg | Neg | MTBC - | | | | | | | | | IS |
| 276 | 239 | 57 | MTB - | MTB - | | Neg | Neg | Neg | Neg | MTBC - | | | | | | | | | IS |
| 277 | 240 | 65 | MTB - | MTB - | | Neg | Neg | Neg | Neg | MTBC - | | | | | | | | | IS |
| 278 | 241 | 65 | MTB - | MTB - | | Neg | Neg | NTM - MAC | NTM | MTBC - | | | | | | | | | IS |
| 279 | 242 | 61 | MTB - | MTB - | | Neg | Neg | Neg | Neg | MTBC - | | | | | | | | | IS |
| 280 | 243 | 64 | MTB - | MTB - | | Neg | Neg | Neg | Neg | MTBC - | | | | | | | | | IS |
| 281 | 244 | 81 | MTB - | MTB - | | Neg | Neg | Neg | Neg | MTBC - | | | | | | | | | IS |
| 282 | 245 | 18 | MTB + / RIF - | MTB + | RIF - | 4+ | Pos | MTB + | Pos | MTBC + | S | S | S | S | S | S | | | IS |
| 283 | 246 | 58 | MTB + / RIF - | MTB + | RIF - | 2+ | Pos | MTB + | Pos | MTBC + | S | S | S | S | S | S | | | IS |
| 284 | 247 | 75 | MTB - | MTB - | | Neg | Neg | Neg | Neg | MTBC - | | | | | | | | | IS |
| 285 | 248 | 47 | MTB - | MTB - | | Neg | Neg | Neg | Neg | MTBC - | | | | | | | | | IS |
| 286 | 249 | 45 | MTB - | MTB - | | Neg | Neg | Neg | Neg | MTBC - | | | | | | | | | IS |
| 287 | 250 | 68 | MTB - | MTB - | | Neg | Neg | Neg | Neg | MTBC - | | | | | | | | | IS |
| 288 | 251 | 48 | MTB - | MTB - | | Neg | Neg | Neg | Neg | MTBC - | | | | | | | | | IS |
| 289 | 252 | 53 | MTB - | MTB - | | Neg | Neg | Neg | Neg | MTBC - | | | | | | | | | IS |
| 290 | 253 | 41 | MTB - | MTB - | | Neg | Neg | Neg | Neg | MTBC - | | | | | | | | | ES |
| 291 | 254 | 40 | MTB - | MTB - | | Neg | Neg | Neg | Neg | MTBC - | | | | | | | | | IS |
| 292 | 255 | 74 | MTB - | MTB - | | 1+ | Pos | Neg | Neg | MTBC - | | | | | | | | | IS |
| 293 | 256 | 66 | MTB - | MTB - | | Neg | Neg | Neg | Neg | MTBC - | | | | | | | | | IS |
| 294 | 257 | 26 | MTB - | MTB - | | Neg | Neg | Neg | Neg | MTBC - | | | | | | | | | IS |
| 295 | 258 | 60 | MTB - | MTB - | | Neg | Neg | Neg | Neg | MTBC - | | | | | | | | | IS |
| 296 | 259 | 28 | MTB - | MTB - | | Neg | Neg | MTB + | Pos | MTBC + | S | S | S | S | S | S | | | IS |
| 297 | 259 | 28 | MTB + / RIF - | MTB + | RIF - | Neg | Neg | MTB + | Pos | MTBC + | S | S | S | S | S | S | | | IS |
| 298 | 260 | 58 | MTB - | MTB - | | Neg | Neg | NTM - M.gordonae | NTM | MTBC - | | | | | | | | | IS |
| 299 | 261 | 46 | MTB + / RIF - | MTB + | RIF - | Neg | Neg | Neg | Neg | MTBC - | | | | | | | | No | IS |
| 300 | 261 | 47 | MTB - | MTB - | | Neg | Neg | Neg | Neg | MTBC - | | | | | | | | | IS |

| | | | | | | | | | | | | | | | | | | | |
|---|---|---|---|---|---|---|---|---|---|---|---|---|---|---|---|---|---|---|---|
| 301 | 262 | 60 | MTB - | MTB - | | Neg | Neg | Neg | Neg | MTBC - | | | | | | | | | IS |
| 302 | 262 | 60 | MTB - | MTB - | | Neg | Neg | Neg | Neg | MTBC - | | | | | | | | | IS |
| 303 | 262 | 60 | MTB - | MTB - | | Neg | Neg | Neg | Neg | MTBC - | | | | | | | | | IS |
| 304 | 263 | 76 | MTB - | MTB - | | Neg | Neg | Neg | Neg | MTBC - | | | | | | | | | IS |
| 305 | 264 | 46 | MTB - | MTB - | | Neg | Neg | NTM - rapid grower | NTM | MTBC - | | | | | | | | | IS |
| 306 | 265 | 77 | MTB + / RIF - | MTB + | RIF - | 3+ | Pos | MTB + | Pos | MTBC + | S | S | S | S | S | S | | | IS |
| 307 | 266 | 72 | MTB - | MTB - | | Neg | Neg | Neg | Neg | MTBC - | | | | | | | | | IS |
| 308 | 266 | 72 | MTB - | MTB - | | Neg | Neg | Neg | Neg | MTBC - | | | | | | | | | IS |
| 309 | 267 | 26 | MTB - | MTB - | | Neg | Neg | Neg | Neg | MTBC - | | | | | | | | | IS |
| 310 | 267 | 26 | MTB - | MTB - | | Neg | Neg | NTM - other M.species | NTM | MTBC - | | | | | | | | | IS |
| 311 | 268 | 31 | MTB - | MTB - | | Neg | Neg | Neg | Neg | MTBC - | | | | | | | | | IS |
| 312 | 269 | 61 | MTB + / RIF - | MTB + | RIF - | 1+ | Pos | MTB + | Pos | MTBC + | S | S | S | S | S | S | | | IS |
| 313 | 270 | 77 | MTB - | MTB - | | Neg | Neg | Neg | Neg | MTBC - | | | | | | | | | IS |
| 314 | 271 | 64 | MTB - | MTB - | | Neg | Neg | Neg | Neg | MTBC - | | | | | | | | | IS |
| 315 | 272 | 65 | MTB - | MTB - | | Neg | Neg | NTM - rapid grower | NTM | MTBC - | | | | | | | | | IS |
| 316 | 273 | 62 | MTB - | MTB - | | Neg | Neg | Neg | Neg | MTBC - | | | | | | | | | IS |
| 317 | 274 | 26 | MTB + / RIF - | MTB + | RIF - | Neg | Neg | MTB + | Pos | MTBC + | S | S | S | S | S | S | | | ES |
| 318 | 275 | 39 | MTB - | MTB - | | Neg | Neg | Neg | Neg | MTBC - | | | | | | | | | IS |
| 319 | 276 | 31 | MTB - | MTB - | | Neg | Neg | Neg | Neg | MTBC - | | | | | | | | | IS |
| 320 | 277 | 59 | MTB - | MTB - | | Neg | Neg | Neg | Neg | MTBC - | | | | | | | | | IS |
| 321 | 278 | 78 | MTB - | MTB - | | Neg | Neg | Neg | Neg | MTBC - | | | | | | | | | IS |
| 322 | 279 | 59 | MTB - | MTB - | | Neg | Neg | NTM - other M.species | NTM | MTBC - | | | | | | | | | IS |
| 323 | 279 | 59 | MTB - | MTB - | | Neg | Neg | Neg | Neg | MTBC - | | | | | | | | | IS |
| 324 | 279 | 59 | MTB - | MTB - | | Neg | Neg | Neg | Neg | MTBC - | | | | | | | | | IS |
| 325 | 280 | 72 | MTB + / RIF - | MTB + | RIF - | 1+ | Pos | Neg | Neg | MTBC - | | | | | | | | Yes | IS |
| 326 | 281 | 50 | MTB - | MTB - | | Neg | Neg | Neg | Neg | MTBC - | | | | | | | | | IS |
| 327 | 282 | 53 | MTB - | MTB - | | Neg | Neg | Neg | Neg | MTBC - | | | | | | | | | IS |
| 328 | 283 | 45 | MTB - | MTB - | | Neg | Neg | Neg | Neg | MTBC - | | | | | | | | | IS |
| 329 | 284 | 36 | MTB - | MTB - | | Neg | Neg | Neg | Neg | MTBC - | | | | | | | | | ES |
| 330 | 285 | 32 | MTB - | MTB - | | Neg | Neg | Neg | Neg | MTBC - | | | | | | | | | ES |
| 331 | 286 | 53 | MTB - | MTB - | | Neg | Neg | Neg | Neg | MTBC - | | | | | | | | | ES |
| 332 | 287 | 47 | MTB + / RIF - | MTB + | RIF - | 1+ | Pos | MTB + | Pos | MTBC + | S | S | S | S | S | S | | | IS |
| 333 | 288 | 51 | MTB - | MTB - | | Neg | Neg | Neg | Neg | MTBC - | | | | | | | | | IS |
| 334 | 289 | 81 | MTB - | MTB - | | Neg | Neg | Neg | Neg | MTBC - | | | | | | | | | IS |
| 335 | 290 | 56 | MTB - | MTB - | | Neg | Neg | Neg | Neg | MTBC - | | | | | | | | | IS |
| 336 | 291 | 52 | MTB - | MTB - | | Neg | Neg | Neg | Neg | MTBC - | | | | | | | | | IS |
| 337 | 292 | 43 | MTB - | MTB - | | Neg | Neg | Neg | Neg | MTBC - | | | | | | | | | IS |
| 338 | 293 | 35 | MTB - | MTB - | | 1+ | Pos | NTM - rapid grower | NTM | MTBC - | | | | | | | | | ES |
| 339 | 294 | 35 | MTB - | MTB - | | Neg | Neg | Neg | Neg | MTBC - | | | | | | | | | IS |
| 340 | 296 | 33 | MTB - | MTB - | | Neg | Neg | Neg | Neg | MTBC - | | | | | | | | | IS |
| 341 | 297 | 31 | MTB - | MTB - | | Neg | Neg | Neg | Neg | MTBC - | | | | | | | | | IS |
| 342 | 298 | 57 | MTB - | MTB - | | Neg | Neg | Neg | Neg | MTBC - | | | | | | | | | IS |
| 343 | 299 | 24 | MTB + / RIF - | MTB + | RIF - | 1+ | Pos | MTB + | Pos | MTBC + | S | S | S | S | S | S | | | IS |

| | | | | | | | | | | | | | | | | | | | |
|---|---|---|---|---|---|---|---|---|---|---|---|---|---|---|---|---|---|---|---|
| 344 | 300 | 47 | MTB - | MTB - | | Neg | Neg | NTM - M.gordonae | NTM | MTBC - | | | | | | | | | IS |
| 345 | 301 | 46 | MTB + / RIF - | MTB + | RIF - | Neg | Neg | MTB + | Pos | MTBC + | S | S | S | S | S | S | | | IS |
| 346 | 302 | 56 | MTB - | MTB - | | Neg | Neg | NTM - MAC | NTM | MTBC - | | | | | | | | | ES |
| 347 | 303 | 60 | MTB - | MTB - | | Neg | Neg | NTM - other M.species | NTM | MTBC - | | | | | | | | | ES |
| 348 | 304 | 25 | MTB - | MTB - | | 1+ | Pos | Neg | Neg | MTBC - | | | | | | | | | IS |
| 349 | 305 | 45 | MTB + / RIF - | MTB + | RIF - | Neg | Neg | MTB + | Pos | MTBC + | S | S | S | S | S | S | | | IS |
| 350 | 306 | 39 | MTB + / RIF - | MTB + | RIF - | Neg | Neg | MTB + | Pos | MTBC + | S | S | S | S | S | S | | | IS |
| 351 | 306 | 39 | MTB - | MTB - | | Neg | Neg | Neg | Neg | MTBC - | | | | | | | | | IS |
| 352 | 307 | 28 | MTB - | MTB - | | Neg | Neg | Neg | Neg | MTBC - | | | | | | | | | IS |
| 353 | 308 | 69 | MTB - | MTB - | | 1+ | Pos | Neg | Neg | MTBC - | | | | | | | | | ES |
| 354 | 309 | 22 | MTB - | MTB - | | Neg | Neg | Neg | Neg | MTBC - | | | | | | | | | IS |
| 355 | 310 | 64 | MTB - | MTB - | | Neg | Neg | Neg | Neg | MTBC - | | | | | | | | | IS |
| 356 | 311 | 63 | MTB - | MTB - | | Neg | Neg | Neg | Neg | MTBC - | | | | | | | | | IS |
| 357 | 312 | 75 | MTB - | MTB - | | Neg | Neg | Neg | Neg | MTBC - | | | | | | | | | IS |
| 358 | 313 | 76 | MTB - | MTB - | | Neg | Neg | Neg | Neg | MTBC - | | | | | | | | | IS |
| 359 | 314 | 14 | MTB - | MTB - | | Neg | Neg | Neg | Neg | MTBC - | | | | | | | | | IS |
| 360 | 315 | 38 | MTB - | MTB - | | Neg | Neg | NTM - MAC | NTM | MTBC - | | | | | | | | | ES |
| 361 | 316 | 32 | MTB - | MTB - | | Neg | Neg | Neg | Neg | MTBC - | | | | | | | | | IS |
| 362 | 317 | 90 | MTB - | MTB - | | Neg | Neg | Neg | Neg | MTBC - | | | | | | | | | IS |
| 363 | 318 | 53 | MTB - | MTB - | | Neg | Neg | NTM - M.gordonae | NTM | MTBC - | | | | | | | | | IS |
| 364 | 319 | 57 | MTB + / RIF - | MTB + | RIF - | 1+ | Pos | MTB + | Pos | MTBC + | S | S | S | S | S | S | | | ES |
| 365 | 320 | 63 | MTB - | MTB - | | Neg | Neg | NTM - M.gordonae | NTM | MTBC - | | | | | | | | | IS |
| 366 | 320 | 63 | MTB - | MTB - | | 1+ | Pos | Neg | Neg | MTBC - | | | | | | | | | IS |
| 367 | 321 | 65 | MTB - | MTB - | | Neg | Neg | Neg | Neg | MTBC - | | | | | | | | | IS |
| 368 | 322 | 32 | MTB - | MTB - | | Neg | Neg | NTM - MAC | NTM | MTBC - | | | | | | | | | IS |
| 369 | 323 | 57 | MTB - | MTB - | | Neg | Neg | Neg | Neg | MTBC - | | | | | | | | | IS |
| 370 | 323 | 57 | MTB - | MTB - | | Neg | Neg | Neg | Neg | MTBC - | | | | | | | | | IS |
| 371 | 323 | 57 | MTB - | MTB - | | Neg | Neg | NTM - M.gordonae | NTM | MTBC - | | | | | | | | | IS |
| 372 | 323 | 57 | MTB - | MTB - | | Neg | Neg | NTM - rapid grower | NTM | MTBC - | | | | | | | | | IS |
| 373 | 323 | 57 | MTB - | MTB - | | Neg | Neg | NTM - rapid grower | NTM | MTBC - | | | | | | | | | IS |
| 374 | 324 | 39 | MTB - | MTB - | | 1+ | Pos | NTM - MAC | NTM | MTBC - | | | | | | | | | ES |
| 375 | 325 | 24 | MTB + / RIF - | MTB + | RIF - | 3+ | Pos | MTB + | Pos | MTBC + | S | S | S | S | S | S | | | IS |
| 376 | 326 | 55 | MTB - | MTB - | | Neg | Neg | MTB + | Pos | MTBC + | S | S | S | S | S | S | | | IS |
| 377 | 326 | 55 | MTB + / RIF - | MTB + | RIF - | Neg | Neg | MTB + | Pos | MTBC + | S | S | S | S | S | S | | | IS |
| 378 | 326 | 55 | MTB - | MTB - | | Neg | Neg | Neg | Neg | MTBC - | | | | | | | | | IS |
| 379 | 327 | 64 | MTB - | MTB - | | Neg | Neg | NTM - M.gordonae | NTM | MTBC - | | | | | | | | | IS |
| 380 | 328 | 24 | MTB - | MTB - | | Neg | Neg | NTM - MAC | NTM | MTBC - | | | | | | | | | IS |
| 381 | 329 | 54 | MTB - | MTB - | | Neg | Neg | Neg | Neg | MTBC - | | | | | | | | | IS |
| 382 | 330 | 51 | MTB - | MTB - | | Neg | Neg | Neg | Neg | MTBC - | | | | | | | | | IS |
| 383 | 331 | 65 | MTB - | MTB - | | Neg | Neg | Neg | Neg | MTBC - | | | | | | | | | IS |
| 384 | 332 | 25 | MTB - | MTB - | | Neg | Neg | Neg | Neg | MTBC - | | | | | | | | | IS |
| 385 | 333 | 35 | MTB - | MTB - | | Neg | Neg | Neg | Neg | MTBC - | | | | | | | | | IS |
| 386 | 334 | 72 | MTB - | MTB - | | Neg | Neg | Neg | Neg | MTBC - | | | | | | | | | IS |

| | | | | | | | | | | | | | | | | | | | |
|---|---|---|---|---|---|---|---|---|---|---|---|---|---|---|---|---|---|---|---|
| 387 | 335 | 52 | MTB - | MTB - | | Neg | Neg | Neg | Neg | MTBC - | | | | | | | | | IS |
| 388 | 336 | 25 | MTB - | MTB - | | Neg | Neg | Neg | Neg | MTBC - | | | | | | | | | IS |
| 389 | 337 | 18 | MTB - | MTB - | | Neg | Neg | Neg | Neg | MTBC - | | | | | | | | | IS |
| 390 | 338 | 62 | MTB - | MTB - | | Neg | Neg | Neg | Neg | MTBC - | | | | | | | | | ES |
| 391 | 339 | 56 | MTB + / RIF - | MTB + | RIF - | 2+ | Pos | MTB + | Pos | MTBC + | S | S | S | S | S | S | | | IS |
| 392 | 339 | 57 | MTB + / RIF - | MTB + | RIF - | 3+ | Pos | MTB + | Pos | MTBC + | S | S | S | S | S | S | | | IS |
| 393 | 340 | 62 | MTB - | MTB - | | Neg | Neg | Neg | Neg | MTBC - | | | | | | | | | IS |
| 394 | 341 | 33 | MTB - | MTB - | | Neg | Neg | Neg | Neg | MTBC - | | | | | | | | | IS |
| 395 | 342 | 8 | MTB - | MTB - | | Neg | Neg | Neg | Neg | MTBC - | | | | | | | | | IS |
| 396 | 343 | 44 | MTB - | MTB - | | Neg | Neg | Neg | Neg | MTBC - | | | | | | | | | IS |
| 397 | 344 | 53 | MTB - | MTB - | | Neg | Neg | Neg | Neg | MTBC - | | | | | | | | | IS |
| 398 | 345 | 54 | MTB - | MTB - | | Neg | Neg | Neg | Neg | MTBC - | | | | | | | | | IS |
| 399 | 346 | 62 | MTB - | MTB - | | Neg | Neg | Neg | Neg | MTBC - | | | | | | | | | IS |
| 400 | 347 | 60 | MTB - | MTB - | | Neg | Neg | Neg | Neg | MTBC - | | | | | | | | | IS |
| 401 | 348 | 59 | MTB - | MTB - | | Neg | Neg | Neg | Neg | MTBC - | | | | | | | | | IS |
| 402 | 348 | 59 | MTB - | MTB - | | Neg | Neg | NTM - other M.species | NTM | MTBC - | | | | | | | | | ES |
| 403 | 349 | 21 | MTB - | MTB - | | Neg | Neg | Neg | Neg | MTBC - | | | | | | | | | IS |
| 404 | 350 | 19 | MTB + / RIF - | MTB + | RIF - | 4+ | Pos | MTB + | Pos | MTBC + | S | S | S | S | S | S | | | ES |
| 405 | 351 | 61 | MTB - | MTB - | | 1+ | Pos | NTM - M.gordonae | NTM | MTBC - | | | | | | | | | ES |
| 406 | 352 | 43 | MTB + / RIF - | MTB + | RIF - | 2+ | Pos | MTB + | Pos | MTBC + | S | R | R | S | S | S | | | IS |
| 407 | 352 | 43 | MTB + / RIF - | MTB + | RIF - | 2+ | Pos | MTB + | Pos | MTBC + | S | R | R | S | S | S | | | IS |
| 408 | 353 | 32 | MTB - | MTB - | | Neg | Neg | NTM - MAC | NTM | MTBC - | | | | | | | | | IS |
| 409 | 353 | 32 | MTB - | MTB - | | Neg | Neg | Neg | Neg | MTBC - | | | | | | | | | IS |
| 410 | 354 | 74 | MTB - | MTB - | | Neg | Neg | Neg | Neg | MTBC - | | | | | | | | | IS |
| 411 | 354 | 74 | MTB - | MTB - | | Neg | Neg | Neg | Neg | MTBC - | | | | | | | | | IS |
| 412 | 355 | 33 | MTB - | MTB - | | Neg | Neg | Neg | Neg | MTBC - | | | | | | | | | IS |
| 413 | 356 | 19 | MTB + / RIF - | MTB + | RIF - | 4+ | Pos | MTB + | Pos | MTBC + | S | S | S | S | S | S | | | ES |
| 414 | 356 | 19 | MTB + / RIF - | MTB + | RIF - | 1+ | Pos | Neg | Neg | MTBC - | | | | | | | | Yes | IS |
| 415 | 357 | 55 | MTB - | MTB - | | Neg | Neg | Neg | Neg | MTBC - | | | | | | | | | IS |
| 416 | 358 | 22 | MTB - | MTB - | | Neg | Neg | Neg | Neg | MTBC - | | | | | | | | | IS |
| 417 | 359 | 50 | MTB - | MTB - | | Neg | Neg | Neg | Neg | MTBC - | | | | | | | | | IS |
| 418 | 360 | 40 | MTB - | MTB - | | Neg | Neg | NTM - MAC | NTM | MTBC - | | | | | | | | | IS |
| 419 | 361 | 54 | MTB - | MTB - | | Neg | Neg | Neg | Neg | MTBC - | | | | | | | | | IS |
| 420 | 362 | 47 | MTB - | MTB - | | Neg | Neg | NTM - MAC | NTM | MTBC - | | | | | | | | | ES |
| 421 | 363 | 41 | MTB - | MTB - | | Neg | Neg | Neg | Neg | MTBC - | | | | | | | | | ES |
| 422 | 364 | 78 | MTB - | MTB - | | Neg | Neg | NTM - other M.species | NTM | MTBC - | | | | | | | | | IS |
| 423 | 364 | 78 | MTB + / RIF - | MTB + | RIF - | Neg | Neg | NTM - rapid grower | NTM | MTBC - | | | | | | | | No | IS |
| 424 | 364 | 78 | MTB - | MTB - | | Neg | Neg | NTM - other M.species | NTM | MTBC - | | | | | | | | | IS |
| 425 | 365 | 49 | MTB - | MTB - | | Neg | Neg | Neg | Neg | MTBC - | | | | | | | | | IS |
| 426 | 367 | 75 | MTB - | MTB - | | Neg | Neg | Neg | Neg | MTBC - | | | | | | | | | IS |
| 427 | 368 | 21 | MTB - | MTB - | | Neg | Neg | Neg | Neg | MTBC - | | | | | | | | | IS |
| 428 | 369 | 45 | MTB - | MTB - | | Neg | Neg | Neg | Neg | MTBC - | | | | | | | | | IS |
| 429 | 370 | 69 | MTB - | MTB - | | Neg | Neg | Neg | Neg | MTBC - | | | | | | | | | IS |

| | | | | | | | | | | | | | | | | | | | |
|---|---|---|---|---|---|---|---|---|---|---|---|---|---|---|---|---|---|---|---|
| 430 | 371 | 65 | MTB - | MTB - | | Neg | Neg | Neg | Neg | MTBC - | | | | | | | | | IS |
| 431 | 372 | 64 | MTB - | MTB - | | Neg | Neg | Neg | Neg | MTBC - | | | | | | | | | IS |
| 432 | 373 | 58 | MTB - | MTB - | | Neg | Neg | Neg | Neg | MTBC - | | | | | | | | | IS |
| 433 | 373 | 59 | MTB - | MTB - | | Neg | Neg | NTM - MAC | NTM | MTBC - | | | | | | | | | IS |
| 434 | 374 | 36 | MTB - | MTB - | | Neg | Neg | Neg | Neg | MTBC - | | | | | | | | | IS |
| 435 | 375 | 56 | MTB + / RIF - | MTB + | RIF - | Neg | Neg | MTB + | Pos | MTBC + | S | S | S | S | S | S | | | IS |
| 436 | 375 | 56 | MTB - | MTB - | | Neg | Neg | MTB + | Pos | MTBC + | S | S | S | S | S | S | | | IS |
| 437 | 376 | 77 | MTB - | MTB - | | Neg | Neg | Neg | Neg | MTBC - | | | | | | | | | IS |
| 438 | 377 | 76 | MTB - | MTB - | | Neg | Neg | Neg | Neg | MTBC - | | | | | | | | | IS |
| 439 | 378 | 28 | MTB + / RIF + | MTB + | RIF + | 2+ | Pos | MTB + | Pos | MTBC + | R | R | R | R | R | R | 531TTG mutation c | | ES |
| 440 | 379 | 46 | MTB - | MTB - | | Neg | Neg | MTB + | Pos | MTBC + | S | S | S | S | S | S | | | IS |
| 441 | 379 | 46 | MTB - | MTB - | | 1+ | Pos | Neg | Neg | MTBC - | | | | | | | | | ES |
| 442 | 380 | 32 | MTB - | MTB - | | Neg | Neg | Neg | Neg | MTBC - | | | | | | | | | ES |
| 443 | 380 | 32 | MTB - | MTB - | | Neg | Neg | Neg | Neg | MTBC - | | | | | | | | | ES |
| 444 | 381 | 15 | MTB + / RIF - | MTB + | RIF - | Neg | Neg | MTB + | Pos | MTBC + | S | S | S | S | S | S | | | IS |
| 445 | 382 | 61 | MTB - | MTB - | | 1+ | Pos | NTM - rapid grower | NTM | MTBC - | | | | | | | | | ES |
| 446 | 383 | 67 | MTB - | MTB - | | Neg | Neg | Neg | Neg | MTBC - | | | | | | | | | IS |
| 447 | 384 | 36 | MTB - | MTB - | | Neg | Neg | Neg | Neg | MTBC - | | | | | | | | | IS |
| 448 | 385 | 50 | MTB - | MTB - | | Neg | Neg | NTM - MAC | NTM | MTBC - | | | | | | | | | IS |
| 449 | 385 | 50 | MTB - | MTB - | | 1+ | Pos | NTM - M.gordonae | NTM | MTBC - | | | | | | | | | IS |
| 450 | 386 | 69 | MTB - | MTB - | | Neg | Neg | Neg | Neg | MTBC - | | | | | | | | | IS |
| 451 | 387 | 50 | MTB - | MTB - | | Neg | Neg | Neg | Neg | MTBC - | | | | | | | | | IS |
| 452 | 387 | 50 | MTB - | MTB - | | 1+ | Pos | Neg | Neg | MTBC - | | | | | | | | | IS |
| 453 | 388 | 30 | MTB - | MTB - | | Neg | Neg | Neg | Neg | MTBC - | | | | | | | | | IS |
| 454 | 389 | 67 | MTB - | MTB - | | Neg | Neg | Neg | Neg | MTBC - | | | | | | | | | IS |
| 455 | 389 | 67 | MTB - | MTB - | | 1+ | Pos | Neg | Neg | MTBC - | | | | | | | | | IS |
| 456 | 390 | 24 | MTB - | MTB - | | Neg | Neg | Neg | Neg | MTBC - | | | | | | | | | IS |
| 457 | 391 | 53 | MTB - | MTB - | | Neg | Neg | Neg | Neg | MTBC - | | | | | | | | | IS |
| 458 | 392 | 34 | MTB - | MTB - | | 1+ | Pos | Neg | Neg | MTBC - | | | | | | | | | ES |
| 459 | 392 | 34 | MTB - | MTB - | | Neg | Neg | Neg | Neg | MTBC - | | | | | | | | | ES |
| 460 | 392 | 34 | MTB - | MTB - | | Neg | Neg | NTM - other M.species | NTM | MTBC - | | | | | | | | | IS |
| 461 | 393 | 25 | MTB + / RIF - | MTB + | RIF - | Neg | Neg | Neg | Neg | MTBC - | | | | | | | | Yes | IS |
| 462 | 394 | 30 | MTB - | MTB - | | Neg | Neg | Neg | Neg | MTBC - | | | | | | | | | IS |
| 463 | 395 | 30 | MTB + / RIF - | MTB + | RIF - | 4+ | Pos | MTB + | Pos | MTBC + | S | S | S | S | S | S | | | IS |
| 464 | 396 | 42 | MTB - | MTB - | | Neg | Neg | Neg | Neg | MTBC - | | | | | | | | | IS |
| 465 | 397 | 88 | MTB - | MTB - | | Neg | Neg | Neg | Neg | MTBC - | | | | | | | | | IS |
| 466 | 398 | 48 | MTB - | MTB - | | Neg | Neg | NTM - M.gordonae | NTM | MTBC - | | | | | | | | | ES |
| 467 | 399 | 59 | MTB - | MTB - | | 1+ | Pos | Neg | Neg | MTBC - | | | | | | | | | IS |
| 468 | 399 | 59 | MTB - | MTB - | | 1+ | Pos | Neg | Neg | MTBC - | | | | | | | | | IS |
| 469 | 399 | 59 | MTB - | MTB - | | 1+ | Pos | NTM - M.gordonae | NTM | MTBC - | | | | | | | | | IS |
| 470 | 400 | 79 | MTB + / RIF - | MTB + | RIF - | 1+ | Pos | MTB + | Pos | MTBC + | S | S | S | S | S | S | | | IS |
| 471 | 401 | 87 | MTB - | MTB - | | Neg | Neg | Neg | Neg | MTBC - | | | | | | | | | IS |
| 472 | 402 | 19 | MTB + / RIF - | MTB + | RIF - | Neg | Neg | Neg | Neg | MTBC - | | | | | | | | Yes | IS |

| | | | | | | | | | | | | | | | | | | | |
|---|---|---|---|---|---|---|---|---|---|---|---|---|---|---|---|---|---|---|---|
| 473 | 403 | 37 | MTB - | MTB - | | Neg | Neg | Neg | Neg | MTBC - | | | | | | | | | IS |
| 474 | 404 | 51 | MTB - | MTB - | | Neg | Neg | Neg | Neg | MTBC - | | | | | | | | | IS |
| 475 | 405 | 44 | MTB + / RIF - | MTB + | RIF - | 4+ | Pos | MTB + | Pos | MTBC + | S | S | S | S | S | S | | | IS |
| 476 | 406 | 83 | MTB - | MTB - | | Neg | Neg | Neg | Neg | MTBC - | | | | | | | | | IS |
| 477 | 406 | 83 | MTB - | MTB - | | 2+ | Pos | Neg | Neg | MTBC - | | | | | | | | | IS |
| 478 | 407 | 58 | MTB - | MTB - | | Neg | Neg | NTM - MAC | NTM | MTBC - | | | | | | | | | IS |
| 479 | 407 | 58 | MTB - | MTB - | | 1+ | Pos | Neg | Neg | MTBC - | | | | | | | | | IS |
| 480 | 408 | 39 | MTB - | MTB - | | Neg | Neg | NTM - MAC | NTM | MTBC - | | | | | | | | | IS |
| 481 | 409 | 39 | MTB - | MTB - | | Neg | Neg | Neg | Neg | MTBC - | | | | | | | | | IS |
| 482 | 410 | 48 | MTB - | MTB - | | 1+ | Pos | Neg | Neg | MTBC - | | | | | | | | | IS |
| 483 | 410 | 48 | MTB - | MTB - | | 1+ | Pos | Neg | Neg | MTBC - | | | | | | | | | ES |
| 484 | 410 | 49 | MTB - | MTB - | | 1+ | Pos | NTM - rapid grower | NTM | MTBC - | | | | | | | | | ES |
| 485 | 411 | 57 | MTB - | MTB - | | Neg | Neg | Neg | Neg | MTBC - | | | | | | | | | IS |
| 486 | 412 | 79 | MTB - | MTB - | | Neg | Neg | Neg | Neg | MTBC - | | | | | | | | | IS |
| 487 | 413 | 8 | MTB - | MTB - | | Neg | Neg | Neg | Neg | MTBC - | | | | | | | | | IS |
| 488 | 414 | 54 | MTB - | MTB - | | Neg | Neg | Neg | Neg | MTBC - | | | | | | | | | IS |
| 489 | 414 | 54 | MTB - | MTB - | | Neg | Neg | NTM - rapid grower | NTM | MTBC - | | | | | | | | | IS |
| 490 | 415 | 23 | MTB - | MTB - | | 1+ | Pos | NTM - rapid grower | NTM | MTBC - | | | | | | | | | ES |
| 491 | 415 | 23 | MTB - | MTB - | | Neg | Neg | NTM - rapid grower | NTM | MTBC - | | | | | | | | | ES |
| 492 | 416 | 72 | MTB - | MTB - | | Neg | Neg | Neg | Neg | MTBC - | | | | | | | | | IS |
| 493 | 416 | 72 | MTB - | MTB - | | Neg | Neg | NTM - rapid grower | NTM | MTBC - | | | | | | | | | IS |
| 494 | 417 | 54 | MTB - | MTB - | | Neg | Neg | NTM - MAC | NTM | MTBC - | | | | | | | | | ES |
| 495 | 418 | 59 | MTB - | MTB - | | Neg | Neg | Neg | Neg | MTBC - | | | | | | | | | IS |
| 496 | 419 | 64 | MTB - | MTB - | | Neg | Neg | Neg | Neg | MTBC - | | | | | | | | | IS |
| 497 | 419 | 64 | MTB - | MTB - | | 1+ | Pos | Neg | Neg | MTBC - | | | | | | | | | IS |
| 498 | 420 | 28 | MTB - | MTB - | | Neg | Neg | NTM - MAC | NTM | MTBC - | | | | | | | | | ES |
| 499 | 421 | 29 | MTB - | MTB - | | Neg | Neg | NTM - MAC | NTM | MTBC - | | | | | | | | | ES |
| 500 | 422 | 21 | MTB - | MTB - | | Neg | Neg | Neg | Neg | MTBC - | | | | | | | | | IS |
| 501 | 423 | 56 | MTB - | MTB - | | Neg | Neg | Neg | Neg | MTBC - | | | | | | | | | IS |
| 502 | 423 | 56 | MTB - | MTB - | | 1+ | Pos | NTM - rapid grower | NTM | MTBC - | | | | | | | | | IS |
| 503 | 424 | 63 | MTB - | MTB - | | Neg | Neg | Neg | Neg | MTBC - | | | | | | | | | IS |
| 504 | 424 | 63 | MTB - | MTB - | | Neg | Neg | NTM - M.gordonae | NTM | MTBC - | | | | | | | | | IS |
| 505 | 425 | 57 | MTB - | MTB - | | Neg | Neg | Neg | Neg | MTBC - | | | | | | | | | IS |
| 506 | 426 | 62 | MTB - | MTB - | | Neg | Neg | Neg | Neg | MTBC - | | | | | | | | | IS |
| 507 | 427 | 67 | MTB - | MTB - | | Neg | Neg | Neg | Neg | MTBC - | | | | | | | | | IS |
| 508 | 428 | 51 | MTB - | MTB - | | 1+ | Pos | NTM - MAC | NTM | MTBC - | | | | | | | | | IS |
| 509 | 428 | 51 | MTB - | MTB - | | 1+ | Pos | Neg | Neg | MTBC - | | | | | | | | | ES |
| 510 | 429 | 44 | MTB + / RIF - | MTB + | RIF - | Neg | Neg | MTB + | Pos | MTBC + | S | S | S | S | S | S | | | ES |
| 511 | 429 | 44 | MTB + / RIF - | MTB + | RIF - | 1+ | Pos | MTB + | Pos | MTBC + | S | S | S | S | S | S | | | IS |
| 512 | 430 | 40 | MTB + / RIF - | MTB + | RIF - | Neg | Neg | MTB + | Pos | MTBC + | S | S | S | S | S | S | | | IS |
| 513 | 431 | 52 | MTB - | MTB - | | Neg | Neg | Neg | Neg | MTBC - | | | | | | | | | IS |
| 514 | 432 | 40 | MTB - | MTB - | | Neg | Neg | Neg | Neg | MTBC - | | | | | | | | | IS |
| 515 | 433 | 58 | MTB - | MTB - | | Neg | Neg | Neg | Neg | MTBC - | | | | | | | | | IS |

| | | | | | | | | | | | | | | | | | | | |
|---|---|---|---|---|---|---|---|---|---|---|---|---|---|---|---|---|---|---|---|
| 516 | 434 | 50 | MTB - | MTB - | | Neg | Neg | Neg | Neg | MTBC - | | | | | | | | | IS |
| 517 | 435 | 33 | MTB - | MTB - | | Neg | Neg | Neg | Neg | MTBC - | | | | | | | | | IS |
| 518 | 436 | 33 | MTB - | MTB - | | Neg | Neg | Neg | Neg | MTBC - | | | | | | | | | IS |
| 519 | 437 | 47 | MTB - | MTB - | | Neg | Neg | Neg | Neg | MTBC - | | | | | | | | | IS |
| 520 | 438 | 38 | MTB - | MTB - | | Neg | Neg | MTB + | Pos | MTBC + | S | S | S | S | S | S | | | IS |
| 521 | 439 | 53 | MTB - | MTB - | | Neg | Neg | Neg | Neg | MTBC - | | | | | | | | | IS |
| 522 | 440 | 46 | MTB - | MTB - | | Neg | Neg | Neg | Neg | MTBC - | | | | | | | | | IS |
| 523 | 440 | 46 | MTB - | MTB - | | 1+ | Pos | Neg | Neg | MTBC - | | | | | | | | | IS |
| 524 | 441 | 35 | MTB - | MTB - | | 1+ | Pos | Neg | Neg | MTBC - | | | | | | | | | ES |
| 525 | 442 | 53 | MTB - | MTB - | | 1+ | Pos | Neg | Neg | MTBC - | | | | | | | | | ES |
| 526 | 443 | 92 | MTB + / RIF + | MTB + | RIF + | 1+ | Pos | Neg | Neg | MTBC - | | | | | | | Prior silent mutatio | Yes | IS |
| 527 | 444 | 17 | MTB + / RIF - | MTB + | RIF - | 1+ | Pos | MTB + | Pos | MTBC + | S | S | S | S | S | S | | | IS |
| 528 | 445 | 35 | MTB - | MTB - | | Neg | Neg | Neg | Neg | MTBC - | | | | | | | | | IS |
| 529 | 446 | 61 | MTB - | MTB - | | 1+ | Pos | Neg | Neg | MTBC - | | | | | | | | | IS |
| 530 | 447 | 54 | MTB - | MTB - | | 1+ | Pos | Neg | Neg | MTBC - | | | | | | | | | IS |
| 531 | 448 | 65 | MTB - | MTB - | | 4+ | Pos | NTM - MAC | NTM | MTBC - | | | | | | | | | IS |
| 532 | 448 | 65 | MTB - | MTB - | | 4+ | Pos | NTM - MAC | NTM | MTBC - | | | | | | | | | ES |
| 533 | 449 | 25 | MTB - | MTB - | | Neg | Neg | MTB + | Pos | MTBC + | S | S | S | S | S | S | | | IS |
| 534 | 450 | 60 | MTB - | MTB - | | Neg | Neg | Neg | Neg | MTBC - | | | | | | | | | IS |
| 535 | 451 | 33 | MTB - | MTB - | | Neg | Neg | Neg | Neg | MTBC - | | | | | | | | | IS |
| 536 | 451 | 33 | MTB - | MTB - | | 2+ | Pos | Neg | Neg | MTBC - | | | | | | | | | IS |
| 537 | 452 | 18 | MTB + / RIF - | MTB + | RIF - | 1+ | Pos | MTB + | Pos | MTBC + | S | S | S | S | S | S | | | IS |
| 538 | 453 | 45 | MTB - | MTB - | | Neg | Neg | Neg | Neg | MTBC - | | | | | | | | | IS |
| 539 | 454 | 35 | MTB - | MTB - | | Neg | Neg | Neg | Neg | MTBC - | | | | | | | | | IS |
| 540 | 455 | 8 | MTB - | MTB - | | Neg | Neg | Neg | Neg | MTBC - | | | | | | | | | IS |
| 541 | 456 | 50 | MTB + / RIF - | MTB + | RIF - | 3+ | Pos | MTB + | Pos | MTBC + | S | S | S | S | S | S | | | ES |
| 542 | 456 | 50 | MTB - | MTB - | | Neg | Neg | NTM - M.gordonae | NTM | MTBC - | | | | | | | | | ES |
| 543 | 456 | 50 | MTB + / RIF - | MTB + | RIF - | 1+ | Pos | Neg | Neg | MTBC - | | | | | | | | Yes | ES |
| 544 | 457 | 28 | MTB + / RIF - | MTB + | RIF - | 4+ | Pos | MTB + | Pos | MTBC + | S | S | S | S | S | S | | | IS |
| 545 | 458 | 69 | MTB - | MTB - | | Neg | Neg | NTM - M.gordonae | NTM | MTBC - | | | | | | | | | IS |
| 546 | 459 | 47 | MTB - | MTB - | | Neg | Neg | Neg | Neg | MTBC - | | | | | | | | | IS |
| 547 | 460 | 19 | MTB - | MTB - | | Neg | Neg | Neg | Neg | MTBC - | | | | | | | | | IS |
| 548 | 461 | 35 | MTB - | MTB - | | 1+ | Pos | NTM - rapid grower | NTM | MTBC - | | | | | | | | | ES |
| 549 | 462 | 51 | MTB - | MTB - | | 1+ | Pos | Neg | Neg | MTBC - | | | | | | | | | IS |
| 550 | 463 | 61 | MTB - | MTB - | | 1+ | Pos | Neg | Neg | MTBC - | | | | | | | | | IS |
| 551 | 463 | 61 | MTB - | MTB - | | Neg | Neg | NTM - rapid grower | NTM | MTBC - | | | | | | | | | IS |
| 552 | 464 | 73 | MTB + / RIF - | MTB + | RIF - | 1+ | Pos | Neg | Neg | MTBC - | | | | | | | | Yes | IS |
| 553 | 465 | 56 | MTB - | MTB - | | Neg | Neg | Neg | Neg | MTBC - | | | | | | | | | IS |
| 554 | 466 | 33 | MTB - | MTB - | | Neg | Neg | Neg | Neg | MTBC - | | | | | | | | | IS |
| 555 | 466 | 33 | MTB - | MTB - | | 1+ | Pos | Neg | Neg | MTBC - | | | | | | | | | IS |
| 556 | 466 | 34 | MTB - | MTB - | | 1+ | Pos | Neg | Neg | MTBC - | | | | | | | | | IS |
| 557 | 467 | 36 | MTB - | MTB - | | Neg | Neg | Neg | Neg | MTBC - | | | | | | | | | IS |
| 558 | 468 | 54 | MTB + / RIF - | MTB + | RIF - | 4+ | Pos | MTB + | Pos | MTBC + | S | S | S | S | S | S | | | IS |

| | | | | | | | | | | | | | | | | | | | |
|---|---|---|---|---|---|---|---|---|---|---|---|---|---|---|---|---|---|---|---|
| 559 | 469 | 51 | MTB - | MTB - | | 1+ | Pos | Neg | Neg | MTBC - | | | | | | | | | ES |
| 560 | 469 | 51 | MTB - | MTB - | | 1+ | Pos | Neg | Neg | MTBC - | | | | | | | | | IS |
| 561 | 470 | 71 | MTB - | MTB - | | Neg | Neg | Neg | Neg | MTBC - | | | | | | | | | IS |
| 562 | 471 | 16 | MTB + / RIF - | MTB + | RIF - | Neg | Neg | MTB + | Pos | MTBC + | S | S | S | S | S | S | | | IS |
| 563 | 471 | 16 | MTB - | MTB - | | Neg | Neg | Neg | Neg | MTBC - | | | | | | | | | IS |
| 564 | 472 | 54 | MTB - | MTB - | | Neg | Neg | Neg | Neg | MTBC - | | | | | | | | | IS |
| 565 | 473 | 64 | MTB - | MTB - | | Neg | Neg | NTM - rapid grower | NTM | MTBC - | | | | | | | | | IS |
| 566 | 474 | 68 | MTB - | MTB - | | Neg | Neg | NTM - MAC | NTM | MTBC - | | | | | | | | | IS |
| 567 | 475 | 39 | MTB - | MTB - | | Neg | Neg | Neg | Neg | MTBC - | | | | | | | | | IS |
| 568 | 475 | 39 | MTB - | MTB - | | 1+ | Pos | Neg | Neg | MTBC - | | | | | | | | | IS |
| 569 | 476 | 21 | MTB - | MTB - | | Neg | Neg | MTB + | Pos | MTBC + | S | S | S | S | S | S | | | IS |
| 570 | 477 | 67 | MTB - | MTB - | | 1+ | Pos | Neg | Neg | MTBC - | | | | | | | | | IS |
| 571 | 478 | 76 | MTB - | MTB - | | 1+ | Pos | Neg | Neg | MTBC - | | | | | | | | | ES |
| 572 | 479 | 93 | MTB + / RIF - | MTB + | RIF - | 1+ | Pos | MTB + | Pos | MTBC + | S | S | S | S | S | S | | | ES |
| 573 | 480 | 52 | MTB - | MTB - | | 1+ | Pos | Neg | Neg | MTBC - | | | | | | | | | ES |
| 574 | 481 | 53 | MTB - | MTB - | | 1+ | Pos | Neg | Neg | MTBC - | | | | | | | | | IS |
| 575 | 481 | 53 | MTB - | MTB - | | 1+ | Pos | NTM - other M.species | NTM | MTBC - | | | | | | | | | IS |
| 576 | 482 | 59 | MTB - | MTB - | | Neg | Neg | Neg | Neg | MTBC - | | | | | | | | | IS |
| 577 | 482 | 59 | MTB - | MTB - | | Neg | Neg | NTM - other M.species | NTM | MTBC - | | | | | | | | | IS |
| 578 | 483 | 25 | MTB - | MTB - | | Neg | Neg | Neg | Neg | MTBC - | | | | | | | | | IS |
| 579 | 484 | 64 | MTB - | MTB - | | Neg | Neg | Neg | Neg | MTBC - | | | | | | | | | IS |
| 580 | 484 | 64 | MTB - | MTB - | | 2+ | Pos | Neg | Neg | MTBC - | | | | | | | | | IS |
| 581 | 485 | 54 | MTB - | MTB - | | Neg | Neg | Neg | Neg | MTBC - | | | | | | | | | IS |
| 582 | 486 | 39 | MTB - | MTB - | | Neg | Neg | Neg | Neg | MTBC - | | | | | | | | | IS |
| 583 | 487 | 72 | MTB - | MTB - | | Neg | Neg | Neg | Neg | MTBC - | | | | | | | | | IS |
| 584 | 488 | 83 | MTB - | MTB - | | 1+ | Pos | Neg | Neg | MTBC - | | | | | | | | | IS |
| 585 | 488 | 83 | MTB - | MTB - | | Neg | Neg | NTM - other M.species | NTM | MTBC - | | | | | | | | | IS |
| 586 | 488 | 83 | MTB - | MTB - | | Neg | Neg | NTM - rapid grower | NTM | MTBC - | | | | | | | | | IS |
| 587 | 489 | 46 | MTB - | MTB - | | Neg | Neg | Neg | Neg | MTBC - | | | | | | | | | IS |
| 588 | 490 | 55 | MTB - | MTB - | | 1+ | Pos | Neg | Neg | MTBC - | | | | | | | | | IS |
| 589 | 491 | 26 | MTB - | MTB - | | Neg | Neg | Neg | Neg | MTBC - | | | | | | | | | IS |
| 590 | 493 | 24 | MTB - | MTB - | | 1+ | Pos | Neg | Neg | MTBC - | | | | | | | | | IS |
| 591 | 493 | 25 | MTB - | MTB - | | 2+ | Pos | Neg | Neg | MTBC - | | | | | | | | | IS |
| 592 | 494 | 58 | MTB + / RIF - | MTB + | RIF - | 2+ | Pos | M.bovis | Pos | MTBC + | S | S | S | R | S | S | | | IS |
| 593 | 495 | 55 | MTB - | MTB - | | Neg | Neg | Neg | Neg | MTBC - | | | | | | | | | IS |
| 594 | 495 | 55 | MTB - | MTB - | | 1+ | Pos | Neg | Neg | MTBC - | | | | | | | | | IS |
| 595 | 496 | 21 | MTB - | MTB - | | Neg | Neg | Neg | Neg | MTBC - | | | | | | | | | IS |
| 596 | 497 | 41 | MTB + / RIF - | MTB + | RIF - | 2+ | Pos | MTB + | Pos | MTBC + | S | S | S | S | S | S | | | IS |
| 597 | 498 | 46 | MTB - | MTB - | | Neg | Neg | Neg | Neg | MTBC - | | | | | | | | | IS |
| 598 | 499 | 59 | MTB - | MTB - | | Neg | Neg | Neg | Neg | MTBC - | | | | | | | | | IS |
| 599 | 499 | 59 | MTB - | MTB - | | 1+ | Pos | Neg | Neg | MTBC - | | | | | | | | | IS |
| 600 | 500 | 59 | MTB + / RIF - | MTB + | RIF - | 4+ | Pos | MTB + | Pos | MTBC + | S | S | S | S | S | S | | | IS |
| 601 | 501 | 72 | MTB - | MTB - | | Neg | Neg | Neg | Neg | MTBC - | | | | | | | | | IS |

| | | | | | | | | | | | | | | | | | | | |
|---|---|---|---|---|---|---|---|---|---|---|---|---|---|---|---|---|---|---|---|
| 602 | 502 | 61 | MTB - | MTB - | | 1+ | Pos | Neg | Neg | MTBC - | | | | | | | | | IS |
| 603 | 503 | 29 | MTB - | MTB - | | Neg | Neg | Neg | Neg | MTBC - | | | | | | | | | IS |
| 604 | 504 | 21 | MTB - | MTB - | | Neg | Neg | Neg | Neg | MTBC - | | | | | | | | | IS |
| 605 | 505 | 39 | MTB - | MTB - | | Neg | Neg | Neg | Neg | MTBC - | | | | | | | | | IS |
| 606 | 506 | 95 | MTB + / RIF - | MTB + | RIF - | 4+ | Pos | MTB + | Pos | MTBC + | S | S | S | S | S | S | | | IS |
| 607 | 507 | 21 | MTB - | MTB - | | Neg | Neg | Neg | Neg | MTBC - | | | | | | | | | IS |
| 608 | 508 | 78 | MTB - | MTB - | | Neg | Neg | Neg | Neg | MTBC - | | | | | | | | | IS |
| 609 | 508 | 78 | MTB - | MTB - | | 1+ | Pos | Neg | Neg | MTBC - | | | | | | | | | IS |
| 610 | 509 | 46 | MTB + / RIF - | MTB + | RIF - | 2+ | Pos | MTB + | Pos | MTBC + | S | S | S | S | S | S | | | IS |
| 611 | 510 | 20 | MTB - | MTB - | | 1+ | Pos | MTB + | Pos | MTBC + | S | S | S | S | S | S | | | ES |
| 612 | 511 | 32 | MTB - | MTB - | | 1+ | Pos | Neg | Neg | MTBC - | | | | | | | | | IS |
| 613 | 512 | 77 | MTB - | MTB - | | Neg | Neg | Neg | Neg | MTBC - | | | | | | | | | IS |
| 614 | 513 | 23 | MTB + / RIF - | MTB + | RIF - | 2+ | Pos | MTB + | Pos | MTBC + | S | S | S | S | S | S | | | IS |
| 615 | 514 | 74 | MTB - | MTB - | | 1+ | Pos | Neg | Neg | MTBC - | | | | | | | | | IS |
| 616 | 515 | 45 | MTB - | MTB - | | Neg | Neg | NTM - MAC | NTM | MTBC - | | | | | | | | | ES |
| 617 | 516 | 37 | MTB - | MTB - | | 1+ | Pos | Neg | Neg | MTBC - | | | | | | | | | IS |
| 618 | 517 | 59 | MTB - | MTB - | | Neg | Neg | Neg | Neg | MTBC - | | | | | | | | | IS |
| 619 | 518 | 83 | MTB - | MTB - | | Neg | Neg | Neg | Neg | MTBC - | | | | | | | | | IS |
| 620 | 518 | 83 | MTB - | MTB - | | 1+ | Pos | Neg | Neg | MTBC - | | | | | | | | | IS |
| 621 | 519 | 74 | MTB - | MTB - | | Neg | Neg | Neg | Neg | MTBC - | | | | | | | | | IS |
| 622 | 520 | 55 | MTB - | MTB - | | 1+ | Pos | Neg | Neg | MTBC - | | | | | | | | | IS |
| 623 | 521 | 44 | MTB - | MTB - | | 1+ | Pos | Neg | Neg | MTBC - | | | | | | | | | IS |
| 624 | 521 | 44 | MTB - | MTB - | | 1+ | Pos | Neg | Neg | MTBC - | | | | | | | | | IS |
| 625 | 521 | 44 | MTB - | MTB - | | Neg | Neg | Neg | Neg | MTBC - | | | | | | | | | IS |
| 626 | 522 | 62 | MTB - | MTB - | | Neg | Neg | Neg | Neg | MTBC - | | | | | | | | | IS |
| 627 | 523 | 49 | MTB - | MTB - | | Neg | Neg | Neg | Neg | MTBC - | | | | | | | | | IS |
| 628 | 524 | 39 | MTB + / RIF - | MTB + | RIF - | 2+ | Pos | MTB + | Pos | MTBC + | S | S | S | S | S | S | | | IS |
| 629 | 525 | 45 | MTB - | MTB - | | Neg | Neg | Neg | Neg | MTBC - | | | | | | | | | IS |
| 630 | 525 | 45 | MTB - | MTB - | | 1+ | Pos | Neg | Neg | MTBC - | | | | | | | | | IS |
| 631 | 526 | 59 | MTB - | MTB - | | Neg | Neg | NTM - M.gordonae | NTM | MTBC - | | | | | | | | | IS |
| 632 | 527 | 52 | MTB - | MTB - | | Neg | Neg | Neg | Neg | MTBC - | | | | | | | | | IS |
| 633 | 528 | 57 | MTB - | MTB - | | Neg | Neg | Neg | Neg | MTBC - | | | | | | | | | IS |
| 634 | 529 | 18 | MTB - | MTB - | | Neg | Neg | Neg | Neg | MTBC - | | | | | | | | | IS |
| 635 | 530 | 30 | MTB - | MTB - | | Neg | Neg | Neg | Neg | MTBC - | | | | | | | | | IS |
| 636 | 531 | 68 | MTB - | MTB - | | Neg | Neg | NTM - other M.species | NTM | MTBC - | | | | | | | | | IS |
| 637 | 531 | 68 | MTB - | MTB - | | 1+ | Pos | NTM - M.gordonae | NTM | MTBC - | | | | | | | | | IS |
| 638 | 532 | 25 | MTB - | MTB - | | Neg | Neg | Neg | Neg | MTBC - | | | | | | | | | IS |
| 639 | 533 | 55 | MTB - | MTB - | | 1+ | Pos | Neg | Neg | MTBC - | | | | | | | | | IS |
| 640 | 534 | 74 | MTB - | MTB - | | 1+ | Pos | NTM - M.gordonae | NTM | MTBC - | | | | | | | | | IS |
| 641 | 535 | 68 | MTB - | MTB - | | Neg | Neg | Neg | Neg | MTBC - | | | | | | | | | IS |
| 642 | 536 | 49 | MTB - | MTB - | | Neg | Neg | Neg | Neg | MTBC - | | | | | | | | | IS |
| 643 | 537 | 56 | MTB - | MTB - | | 2+ | Pos | NTM - M.gordonae | NTM | MTBC - | | | | | | | | | IS |
| 644 | 538 | 67 | MTB - | MTB - | | 1+ | Pos | Neg | Neg | MTBC - | | | | | | | | | IS |

| | | | | | | | | | | | | | | | | | | | |
|---|---|---|---|---|---|---|---|---|---|---|---|---|---|---|---|---|---|---|---|
| 645 | 539 | 52 | MTB + / RIF - | MTB + | RIF - | 4+ | Pos | MTB + | Pos | MTBC + | S | S | S | S | S | S | | | IS |
| 646 | 540 | 43 | MTB - | MTB - | | 1+ | Pos | Neg | Neg | MTBC - | | | | | | | | | IS |
| 647 | 541 | 50 | MTB + / RIF - | MTB + | RIF - | Neg | Neg | MTB + | Pos | MTBC + | S | S | S | S | S | S | | | IS |
| 648 | 542 | 30 | MTB - | MTB - | | Neg | Neg | Neg | Neg | MTBC - | | | | | | | | | ES |
| 649 | 543 | 67 | MTB - | MTB - | | Neg | Neg | Neg | Neg | MTBC - | | | | | | | | | IS |
| 650 | 544 | 57 | MTB - | MTB - | | Neg | Neg | Neg | Neg | MTBC - | | | | | | | | | IS |
| 651 | 545 | 57 | MTB - | MTB - | | 1+ | Pos | Neg | Neg | MTBC - | | | | | | | | | ES |
| 652 | 546 | 29 | MTB - | MTB - | | 1+ | Pos | Neg | Neg | MTBC - | | | | | | | | | IS |
| 653 | 547 | 35 | MTB - | MTB - | | 1+ | Pos | Neg | Neg | MTBC - | | | | | | | | | IS |
| 654 | 548 | 35 | MTB - | MTB - | | 1+ | Pos | Neg | Neg | MTBC - | | | | | | | | | IS |
| 655 | 549 | 58 | MTB - | MTB - | | 2+ | Pos | Neg | Neg | MTBC - | | | | | | | | | ES |
| 656 | 550 | 64 | MTB - | MTB - | | 1+ | Pos | Neg | Neg | MTBC - | | | | | | | | | ES |
| 657 | 551 | 43 | MTB - | MTB - | | 1+ | Pos | Neg | Neg | MTBC - | | | | | | | | | IS |
| 658 | 552 | 23 | MTB + / RIF - | MTB + | RIF - | 2+ | Pos | MTB + | Pos | MTBC + | S | S | S | S | S | S | | | IS |
| 659 | 553 | 50 | MTB - | MTB - | | Neg | Neg | Neg | Neg | MTBC - | | | | | | | | | IS |
| 660 | 554 | 29 | MTB - | MTB - | | Neg | Neg | Neg | Neg | MTBC - | | | | | | | | | IS |
| 661 | 555 | 64 | MTB - | MTB - | | 1+ | Pos | NTM - rapid grower | NTM | MTBC - | | | | | | | | | ES |
| 662 | 556 | 69 | MTB - | MTB - | | 1+ | Pos | Neg | Neg | MTBC - | | | | | | | | | IS |
| 663 | 557 | 22 | MTB - | MTB - | | 1+ | Pos | Neg | Neg | MTBC - | | | | | | | | | ES |
| 664 | 558 | 38 | MTB + / RIF - | MTB + | RIF - | 2+ | Pos | MTB + | Pos | MTBC + | S | S | S | S | S | S | | | IS |
| 665 | 559 | 46 | MTB - | MTB - | | 1+ | Pos | NTM - rapid grower | NTM | MTBC - | | | | | | | | | ES |
| 666 | 560 | 46 | MTB + / RIF - | MTB + | RIF - | 3+ | Pos | MTB + | Pos | MTBC + | S | S | S | S | S | S | | | ES |
| 667 | 561 | 61 | MTB - | MTB - | | Neg | Neg | Neg | Neg | MTBC - | | | | | | | | | IS |
| 668 | 562 | 45 | MTB - | MTB - | | Neg | Neg | Neg | Neg | MTBC - | | | | | | | | | IS |
| 669 | 563 | 28 | MTB - | MTB - | | Neg | Neg | Neg | Neg | MTBC - | | | | | | | | | IS |
| 670 | 564 | 23 | MTB - | MTB - | | Neg | Neg | Neg | Neg | MTBC - | | | | | | | | | IS |
| 671 | 565 | 18 | MTB + / RIF - | MTB + | RIF - | Neg | Neg | MTB + | Pos | MTBC + | S | S | S | S | S | S | | | IS |
| 672 | 566 | 49 | MTB + / RIF - | MTB + | RIF - | 2+ | Pos | MTB + | Pos | MTBC + | S | S | S | S | S | S | | | IS |
| 673 | 567 | 18 | MTB - | MTB - | | Neg | Neg | Neg | Neg | MTBC - | | | | | | | | | IS |
| 674 | 568 | 21 | MTB + / RIF - | MTB + | RIF - | 1+ | Pos | MTB + | Pos | MTBC + | S | S | S | S | S | S | | | IS |
| 675 | 569 | 87 | MTB - | MTB - | | Neg | Neg | Neg | Neg | MTBC - | | | | | | | | | IS |
| 676 | 570 | 71 | MTB - | MTB - | | Neg | Neg | Neg | Neg | MTBC - | | | | | | | | | IS |
| 677 | 571 | 47 | MTB - | MTB - | | Neg | Neg | Neg | Neg | MTBC - | | | | | | | | | IS |
| 678 | 572 | 47 | MTB - | MTB - | | Neg | Neg | Neg | Neg | MTBC - | | | | | | | | | IS |
| 679 | 573 | 43 | MTB - | MTB - | | Neg | Neg | Neg | Neg | MTBC - | | | | | | | | | IS |
| 680 | 574 | 30 | MTB - | MTB - | | Neg | Neg | Neg | Neg | MTBC - | | | | | | | | | IS |
| 681 | 575 | 57 | MTB - | MTB - | | Neg | Neg | Neg | Neg | MTBC - | | | | | | | | | ES |
| 682 | 576 | 25 | MTB + / RIF - | MTB + | RIF - | 3+ | Pos | MTB + | Pos | MTBC + | S | S | S | S | S | S | | | IS |
| 683 | 577 | 82 | MTB - | MTB - | | Neg | Neg | Neg | Neg | MTBC - | | | | | | | | | IS |
| 684 | 577 | 82 | MTB - | MTB - | | 1+ | Pos | Neg | Neg | MTBC - | | | | | | | | | IS |
| 685 | 578 | 48 | MTB - | MTB - | | Neg | Neg | Neg | Neg | MTBC - | | | | | | | | | IS |
| 686 | 579 | 35 | MTB - | MTB - | | 2+ | Pos | Neg | Neg | MTBC - | | | | | | | | | IS |
| 687 | 580 | 54 | MTB + / RIF - | MTB + | RIF - | 2+ | Pos | MTB + | Pos | MTBC + | S | R | R | S | S | S | | | ES |

| | | | | | | | | | | | | | | | | | | | |
|---|---|---|---|---|---|---|---|---|---|---|---|---|---|---|---|---|---|---|---|
| 688 | 581 | 46 | MTB - | MTB - | | Neg | Neg | NTM - other M.species | NTM | MTBC - | | | | | | | | | IS |
| 689 | 582 | 63 | MTB + / RIF - | MTB + | RIF - | 1+ | Pos | MTB + | Pos | MTBC + | S | S | S | S | S | S | | | IS |
| 690 | 583 | 54 | MTB - | MTB - | | 1+ | Pos | Neg | Neg | MTBC - | | | | | | | | | IS |
| 691 | 584 | 62 | MTB - | MTB - | | Neg | Neg | NTM - M.gordonae | NTM | MTBC - | | | | | | | | | IS |
| 692 | 585 | 64 | MTB - | MTB - | | Neg | Neg | Neg | Neg | MTBC - | | | | | | | | | IS |
| 693 | 586 | 79 | MTB + / RIF - | MTB + | RIF - | Neg | Neg | MTB + | Pos | MTBC + | S | S | S | S | S | S | | | IS |
| 694 | 587 | 44 | MTB - | MTB - | | Neg | Neg | Neg | Neg | MTBC - | | | | | | | | | IS |
| 695 | 588 | 57 | MTB - | MTB - | | Neg | Neg | Neg | Neg | MTBC - | | | | | | | | | IS |
| 696 | 589 | 31 | MTB + / RIF - | MTB + | RIF - | 2+ | Pos | MTB + | Pos | MTBC + | S | S | S | S | S | S | | | ES |
| 697 | 590 | 42 | MTB + / RIF + | MTB + | RIF + | 4+ | Pos | MTB + | Pos | MTBC + | S | R | S | S | S | S | Silent mutation (51 | | IS |
| 698 | 591 | 72 | MTB - | MTB - | | Neg | Neg | Neg | Neg | MTBC - | | | | | | | | | IS |
| 699 | 592 | 31 | MTB - | MTB - | | 1+ | Pos | MTB + | Pos | MTBC + | S | S | S | S | S | S | | | ES |
| 700 | 593 | 62 | MTB - | MTB - | | Neg | Neg | Neg | Neg | MTBC - | | | | | | | | | IS |
| 701 | 594 | 54 | MTB - | MTB - | | 2+ | Pos | Neg | Neg | MTBC - | | | | | | | | | IS |
| 702 | 595 | 60 | MTB - | MTB - | | Neg | Neg | Neg | Neg | MTBC - | | | | | | | | | IS |
| 703 | 596 | 43 | MTB - | MTB - | | 1+ | Pos | Neg | Neg | MTBC - | | | | | | | | | ES |
| 704 | 597 | 24 | MTB + / RIF - | MTB + | RIF - | 2+ | Pos | MTB + | Pos | MTBC + | S | S | S | S | S | S | | | IS |
| 705 | 598 | 52 | MTB - | MTB - | | 2+ | Pos | Neg | Neg | MTBC - | | | | | | | | | IS |
| 706 | 599 | 61 | MTB - | MTB - | | Neg | Neg | Neg | Neg | MTBC - | | | | | | | | | IS |
| 707 | 600 | 63 | MTB - | MTB - | | 1+ | Pos | Neg | Neg | MTBC - | | | | | | | | | IS |
| 708 | 601 | 28 | MTB + / RIF - | MTB + | RIF - | 1+ | Pos | MTB + | Pos | MTBC + | S | S | S | S | S | S | | | IS |
| 709 | 602 | 44 | MTB + / RIF - | MTB + | RIF - | 4+ | Pos | MTB + | Pos | MTBC + | S | S | S | S | S | S | | | IS |
| 710 | 604 | 51 | MTB - | MTB - | | Neg | Neg | Neg | Neg | MTBC - | | | | | | | | | IS |
| 711 | 605 | 20 | MTB - | MTB - | | Neg | Neg | Neg | Neg | MTBC - | | | | | | | | | IS |
| 712 | 605 | 20 | MTB - | MTB - | | 2+ | Pos | Neg | Neg | MTBC - | | | | | | | | | IS |
| 713 | 606 | 37 | MTB - | MTB - | | 1+ | Pos | Neg | Neg | MTBC - | | | | | | | | | ES |
| 714 | 607 | 58 | MTB - | MTB - | | 1+ | Pos | Neg | Neg | MTBC - | | | | | | | | | IS |
| 715 | 608 | 68 | MTB - | MTB - | | 2+ | Pos | NTM - rapid grower | NTM | MTBC - | | | | | | | | | IS |
| 716 | 609 | 37 | MTB - | MTB - | | 1+ | Pos | Neg | Neg | MTBC - | | | | | | | | | ES |
| 717 | 610 | 22 | MTB - | MTB - | | 1+ | Pos | Neg | Neg | MTBC - | | | | | | | | | ES |
| 718 | 611 | 61 | MTB - | MTB - | | 1+ | Pos | Neg | Neg | MTBC - | | | | | | | | | IS |
| 719 | 612 | 30 | MTB - | MTB - | | 2+ | Pos | Neg | Neg | MTBC - | | | | | | | | | ES |
| 720 | 613 | 84 | MTB - | MTB - | | 1+ | Pos | Neg | Neg | MTBC - | | | | | | | | | IS |
| 721 | 614 | 34 | MTB - | MTB - | | Neg | Neg | Neg | Neg | MTBC - | | | | | | | | | IS |
| 722 | 615 | 40 | MTB - | MTB - | | Neg | Neg | Neg | Neg | MTBC - | | | | | | | | | IS |
| 723 | 616 | 41 | MTB + / RIF - | MTB + | RIF - | 3+ | Pos | Neg | Neg | MTBC - | | | | | | | | Yes | IS |
| 724 | 617 | 53 | MTB - | MTB - | | Neg | Neg | Neg | Neg | MTBC - | | | | | | | | | IS |
| 725 | 618 | 59 | MTB - | MTB - | | Neg | Neg | Neg | Neg | MTBC - | | | | | | | | | IS |
| 726 | 619 | 22 | MTB - | MTB - | | Neg | Neg | Neg | Neg | MTBC - | | | | | | | | | IS |
| 727 | 620 | 42 | MTB - | MTB - | | Neg | Neg | MTB + | Pos | MTBC + | S | S | S | S | S | S | | | IS |
| 728 | 621 | 70 | MTB - | MTB - | | Neg | Neg | NTM - rapid grower | NTM | MTBC - | | | | | | | | | IS |
| 729 | 622 | 65 | MTB - | MTB - | | Neg | Neg | Neg | Neg | MTBC - | | | | | | | | | IS |
| 730 | 623 | 54 | MTB - | MTB - | | Neg | Neg | Neg | Neg | MTBC - | | | | | | | | | IS |

| | | | | | | | | | | | | | | | | | | | |
|---|---|---|---|---|---|---|---|---|---|---|---|---|---|---|---|---|---|---|---|
| 731 | 624 | 37 | MTB - | MTB - | | Neg | Neg | Neg | Neg | MTBC - | | | | | | | | | IS |
| 732 | 625 | 41 | MTB - | MTB - | | 1+ | Pos | NTM - MAC | NTM | MTBC - | | | | | | | | | IS |
| 733 | 626 | 68 | MTB - | MTB - | | Neg | Neg | NTM - rapid grower | NTM | MTBC - | | | | | | | | | IS |
| 734 | 627 | 74 | MTB - | MTB - | | Neg | Neg | NTM - other M.species | NTM | MTBC - | | | | | | | | | IS |
| 735 | 628 | 39 | MTB + / RIF - | MTB + | RIF - | 3+ | Pos | MTB + | Pos | MTBC + | S | R | S | S | S | S | | | IS |
| 736 | 629 | 33 | MTB - | MTB - | | Neg | Neg | Neg | Neg | MTBC - | | | | | | | | | IS |
| 737 | 630 | 56 | MTB + / RIF - | MTB + | RIF - | Neg | Neg | MTB + | Pos | MTBC + | S | S | S | S | S | S | | | IS |
| 738 | 631 | 42 | MTB - | MTB - | | Neg | Neg | Neg | Neg | MTBC - | | | | | | | | | IS |
| 739 | 632 | 74 | MTB + / RIF - | MTB + | RIF - | 3+ | Pos | MTB + | Pos | MTBC + | S | S | S | S | S | S | | | IS |
| 740 | 633 | 49 | MTB - | MTB - | | Neg | Neg | Neg | Neg | MTBC - | | | | | | | | | IS |
| 741 | 634 | 27 | MTB + / RIF - | MTB + | RIF - | 4+ | Pos | MTB + | Pos | MTBC + | S | S | S | S | S | S | | | ES |
| 742 | 635 | 34 | MTB - | MTB - | | Neg | Neg | Neg | Neg | MTBC - | | | | | | | | | IS |
| 743 | 636 | 53 | MTB - | MTB - | | Neg | Neg | Neg | Neg | MTBC - | | | | | | | | | IS |
| 744 | 637 | 26 | MTB + / RIF - | MTB + | RIF - | 3+ | Pos | MTB + | Pos | MTBC + | S | S | S | S | S | S | | | ES |
| 745 | 638 | 59 | MTB - | MTB - | | Neg | Neg | Neg | Neg | MTBC - | | | | | | | | | IS |
| 746 | 639 | 62 | MTB - | MTB - | | Neg | Neg | Neg | Neg | MTBC - | | | | | | | | | IS |
| 747 | 640 | 41 | MTB - | MTB - | | Neg | Neg | Neg | Neg | MTBC - | | | | | | | | | IS |
| 748 | 641 | 88 | MTB - | MTB - | | Neg | Neg | Neg | Neg | MTBC - | | | | | | | | | IS |
| 749 | 642 | 32 | MTB - | MTB - | | Neg | Neg | Neg | Neg | MTBC - | | | | | | | | | IS |
| 750 | 643 | 36 | MTB - | MTB - | | Neg | Neg | Neg | Neg | MTBC - | | | | | | | | | IS |
| 751 | 644 | 48 | MTB + / RIF - | MTB + | RIF - | 2+ | Pos | MTB + | Pos | MTBC + | S | S | S | S | S | S | | | IS |
